# Crystal structures of two dioxomolybdenum complexes stabilized by salan ligands featuring phenyl and cyclo­hexyl backbones

**DOI:** 10.1107/S2056989022000524

**Published:** 2022-02-01

**Authors:** Tristhan Trieu-Tran, Stephenie N. Martinez, Jacob P. Brannon, S. Chantal E. Stieber, Alex John

**Affiliations:** a Chemistry & Biochemistry Department, California State Polytechnic University, Pomona, 3801 W. Temple Ave., Pomona, CA 91768, USA

**Keywords:** crystal structure, molybdenum, salan ligand, *cis*-dioxo, DODH

## Abstract

Two *cis*-dioxomolybdenum complexes based on salan ligands with different backbones are reported. The first complex, dioxido{2,2′-[l,2-phenyl­enebis(imino­methyl­ene)]bis­(phenolato)}molybdenum(VI) di­methyl­formamide disolvate, [Mo(C_20_H_18_N_2_O_2_)O_2_]·2C_3_H_7_NO (^Ph^LMoO_2_, **1b**), features a phenyl backbone, while the second complex, (6,6′-{[(cyclo­hexane-1,2-di­yl)bis(aza­nedi­yl)]bis­(methyl­ene)}bis­(2,4-di-*tert*-butyl­phenolato))dioxidomolybdenum(VI) methanol disolvate, [Mo(C_36_H_56_N_2_O_2_)O_2_]·2CH_3_OH (^Cy^LMoO_2_, **2b**), is based on a cyclo­hexyl backbone. These complexes crystallized as solvated species, **1b**·2DMF and **2b**·2MeOH. The salan ligands ^Ph^LH_2_ (**1a**) and ^Cy^LH_2_ (**2a**) coordinate to the molybdenum center in these complexes **1b** and **2b** in a κ^2^ *N*,κ^2^ *O* fashion, forming a distorted octa­hedral geometry. The Mo—N and Mo—O distances are 2.3475 (16) and 1.9567 (16) Å, respectively, in **1b** while the corresponding measurements are Mo—N = 2.3412 (12) Å, and Mo—O = 1.9428 (10) Å for **2b**. A key geometrical feature is that the N—Mo—N angle of 72.40 (4)° in ^Cy^LMoO_2_ is slightly less than that of the ^Ph^LMoO_2_ angle of 75.18 (6)°, which is attributed to the flexibility of the cyclo­hexane ring between the nitro­gen as compared to the rigid phenyl ring in the ^Ph^LMoO_2_.

## Abstract 2

Two *cis*-dioxomolybdenum complexes based on salan ligands with different backbones are reported. The salan ligands coordinate to the molybdenum center in a κ^2^ *N*,κ^2^ *O* fashion, forming a distorted octa­hedral geometry. These complexes crystallized as di­methyl­formamide and methanol solvated species.

## Chemical context

Molybdenum centers are present in the active sites of various enzymes including nitro­genases, sulfite oxidase, xanthine oxidase, and DMSO reductase that catalyze two-electron redox processes (Hille *et al.*, 2014 ▸; Enemark *et al.*, 2004 ▸; Hille, 1996 ▸). This is attributed to the large number of stable oxidation states and coordination environments that can be achieved, as well as the solubility of molybdate salts in water. A majority of these enzymes are referred to as oxo-molybdenum enzymes due to the presence of at least one Mo=O moiety in the active site. The sulfite oxidase family of enzymes contains a *cis*-dioxo molybdenum(VI) (*L_n_* MoO_2_) center in its active site (Hille *et al.*, 2014 ▸). Apart from being studied as models to understand biological systems, oxomolybdenum complexes have also found utility in processes such as olefin metathesis, olefin epoxidation, cytotoxic studies, and cyclic ester polymerizations (Hossain *et al.* 2020 ▸; Mayilmurugan *et al.* 2013 ▸; Yang *et al.* 2007 ▸). Mononuclear molybdenum complexes are generally distinguished by stretching frequencies {*u*(O=Mo=O)} in the 910–950 cm^−1^ and 890–925 cm^−1^ regions, which are characteristic of a *cis*-MoO_2_ fragment (Chakravarthy & Chand, 2011 ▸). A variety of ligand architectures have been successful in stabilizing the oxomol­yb­denum core in these complexes (Ziegler *et al.* 2009 ▸; Subramanian *et al.* 1984 ▸; Rajan *et al.* 1983 ▸). Dioxomolybdenum complexes stabilized by salan ligands have been used extensively for various applications (Roy *et al.*, 2017 ▸; Whiteoak *et al.*, 2009 ▸). The modular nature for the synthesis of salan ligands allows for incorporation of steric and electronic variations in the ligand framework to tune the reactivity of the molybdenum center. We are exploring the utility of dioxomolybdenum complexes in catalyzing the de­oxy­dehydration (DODH) reaction with a focus on understanding ligand effects on catalytic activity. This work reports synthesis and crystal structures of two molybdenum complexes including a crystallographically uncharacterized complex, dioxido[2,2′-{l,2-phenyl­enebis(imino­methyl­ene)bis­(phenolato)]molyb­den­um(VI), ^Ph^LMoO_2_ (**1b**) (Rajan *et al.* 1983 ▸). The second is a known complex with a new unit cell, (Ziegler *et al.*, 2009 ▸), 6,6′-{[(cyclo­hexane-1,2-di­yl)bis­(aza­nedi­yl)]bis­(methyl­ene)}bis­(2,4-di-*tert*-butyl­phenolato))dioxidomolybdenum(VI), ^Cy^LMoO_2_ (**2b**). 
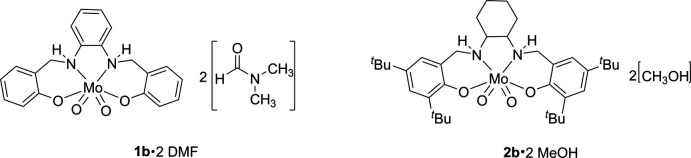


## Structural commentary

**Figure 1:**
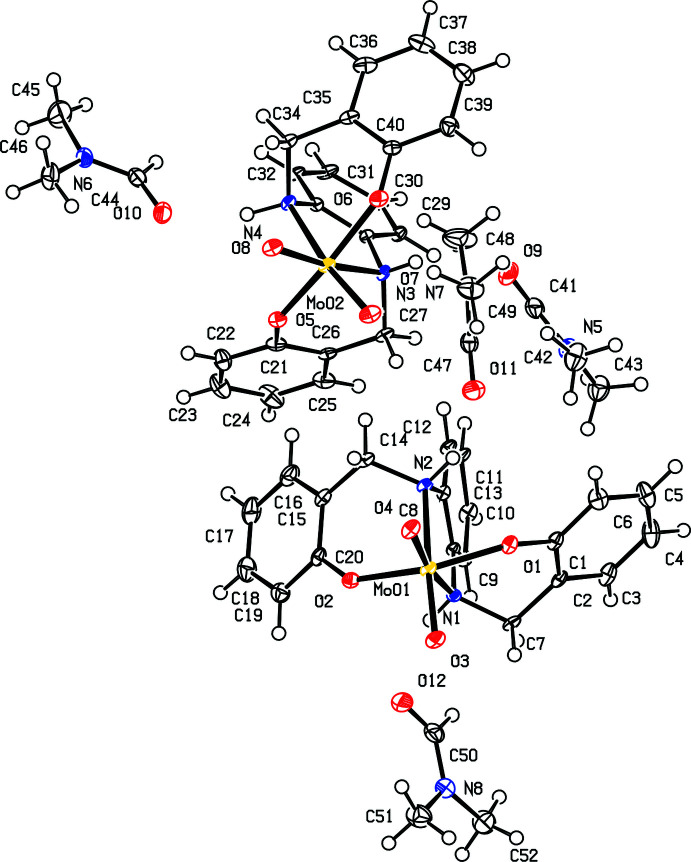
View of 2[^Ph^LMoO_2_]·4[DMF] (**1b**) with 50% probability ellipsoids.

**Figure 2:**
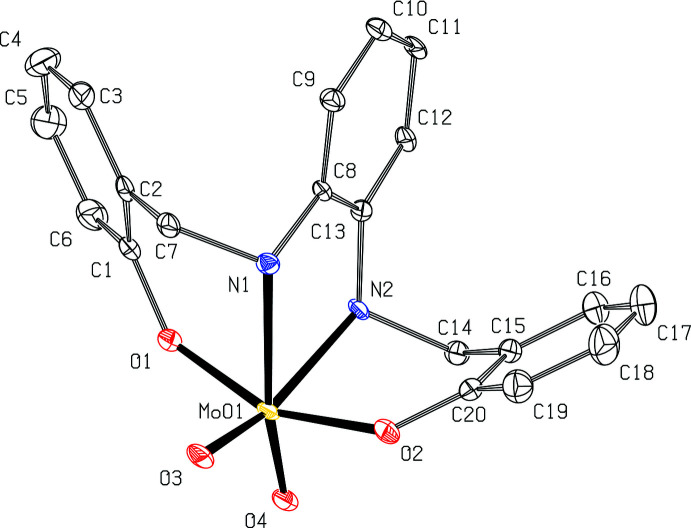
View of one mol­ecule of ^Ph^LMoO_2_ (**1b**) with 50% probability ellipsoids. The DMF mol­ecule and H atoms are omitted for clarity.

The asymmetric unit of ^Ph^LMoO_2_ (**1b**) contains two mol­ecules of ^Ph^LMoO_2_ and four mol­ecules of di­methyl­formamide (DMF), as shown in Fig. 1 ▸. Fig. 2 ▸ shows one mol­ecule of ^Ph^LMoO_2_ with hydrogen atoms and solvent removed for clarity. In this system, the salan ligand ^Ph^LH_2_ (**1a**) coordinates to the molybdenum center in a κ^2^ *N*,κ^2^ *O* fashion, forming a distorted octa­hedral geometry. The angles formed around the molybdenum core are 80.23 (6)° for O1—Mo01—N1, 157.78 (6)° for O1—Mo01—O2, 75.18 (6)° for N1—Mo01—N2, and 109.80 (7)° for O3—Mo01—O4. These angles are consistent with a system that is significantly distorted from octa­hedral geometry with bond angles resulting from the salan ligand ranging from 75.18 (6) to 84.38 (7)°, while the angle between the ‘oxo’ oxygens of 109.80 (7)° is close to the ideal tetra­hedral angle of 109.5°. Analogous bond angles in the second molecule in the unit cell are the same within 0.01 Å. The bond distances between the molybdenum center and ligand atoms for Mo01—N1 and Mo01—O1 are 2.3475 (16) and 1.9567 (16) Å, respectively. The notable bond distances from the salan ligand are O1—C1 at 1.377 (2) Å, N1—C7 at 1.486 (3) Å, C2—C7 at 1.515 (3) Å, N1—C8 at 1.389 (8) Å, and C8—C13 at 1.419 (3) Å. Analogous bond distances in the second molecule in the unit cell are the same within 0.01 Å as distances for O1—C1 and N1—C8, respectively. The other bond distances have variations of 0.2–0.3 Å, with N3—C27 at 1.519 (3) Å, C26—C27 at 1.490 (3) Å, and C28—C33 at 1.392 (3) Å.

**Figure 3:**
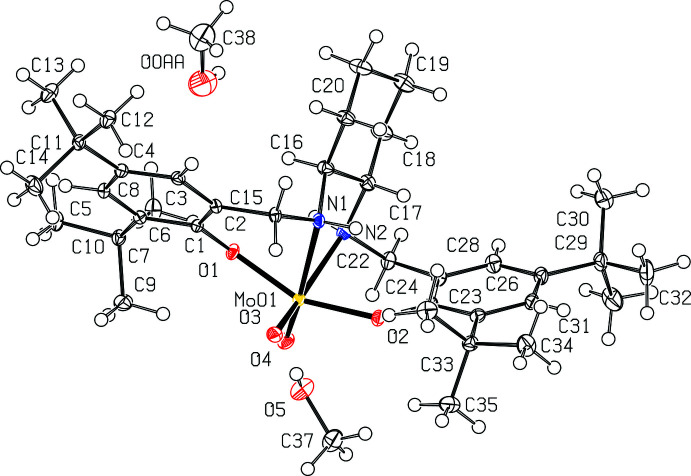
View of one mol­ecule of ^cy^LMoO_2_·2MeOH (**2b**) with 50% probability ellipsoids.

**Figure 4:**
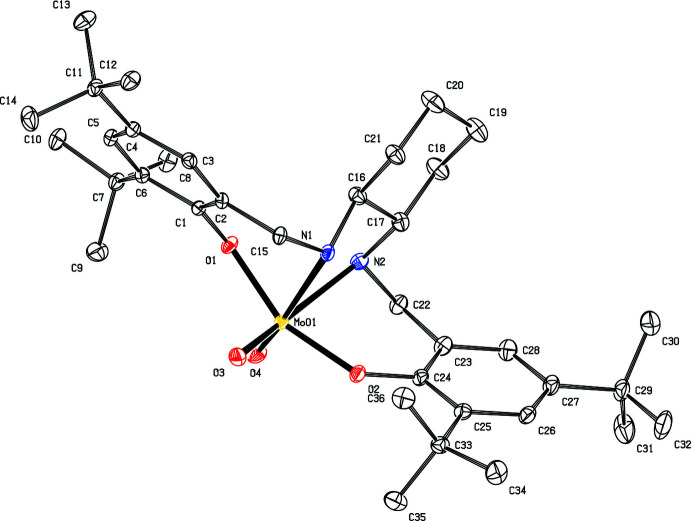
View of one mol­ecule of ^cy^LMoO_2_ (**2b**) with 50% probability ellipsoids. The MeOH mol­ecules and H atoms are omitted for clarity.

The asymmetric unit of ^Cy^LMoO_2_ (**2b**) contains one mol­ecule of ^Cy^LMoO_2_ and two mol­ecules of methanol (MeOH) (Fig. 3 ▸). The salan ligand ^Cy^LH_2_ (**2a**) binds in the same κ^2^ *N*,κ^2^ *O* fashion that complex **1b** does. Fig. 4 ▸ shows ^Cy^LMoO_2_ with the hydrogen atoms removed for clarity. The complex also has a distorted octa­hedral geometry with angles of O3—Mo01—O1 at 96.36 (5)°, O1—Mo01—N1 at 76.73 (4)°, N1—Mo01—N2 at 72.40 (4)°, N2—Mo01—O2 at 78.91 (4)°, O2—Mo01—O4 at 100.19 (5)°, O2—Mo01—O3 at 94.58 (5)°. These angles are between 5 and 10° of the ideal 90° for octa­hedral geometry. The N1—Mo01—N2 angle at 72.40 (4)° is slightly less than that of the ^Ph^LMoO_2_ angle of 75.81 (6)°, which is attributed to the flexibility of the cyclo­hexane ring between the nitro­gen atoms compared to the rigid phenyl ring in the ^Ph^LMoO_2._ Metal–ligand bond distances are found for Mo01—O1 at 1.9428 (10) Å, Mo01—O2 at 1.9484 (10) Å, Mo01—O3 at 1.7125 (10) Å, Mo01—O4 at 1.7226 (11) Å, Mo01—N1 at 2.3412 (12) Å, and Mo01—N2 at 2.3384 (12) Å. Other ligand distances and bond lengths within the phenyl rings are consistent with analagous distances in ^Ph^LMoO_2_ (**1b**). The cylohexane bond distances are consistent with single C—C bonds. The bond lengths observed are not statistically different than those reported by Ziegler *et al.* (2009 ▸). There are a few statistically different angles, specifically around the molybdenum center where Table 1 ▸ shows the correlating bond angles. These bond-angle differences are most likely due to improved *R*1 of 2.78% as compared to the previously reported *R*1 of 5.5% and higher solvent disorder in the reported structure.

## Supra­molecular features

**Figure 5:**
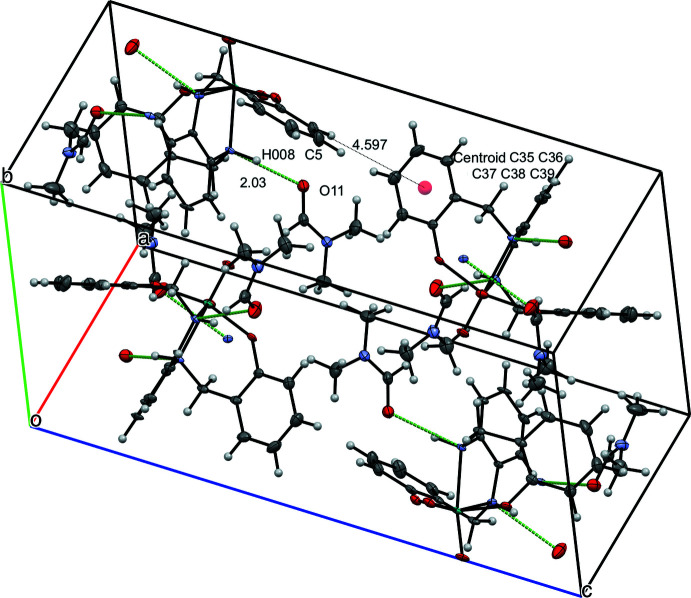
View of six mol­ecules of ^Ph^LMoO_2_ and five mol­ecules of DMF in the unit cell with 50% probability ellipsoids, highlighting inter­molecular distances. Distances between H atoms are listed without standard deviations because the H atoms were positionally fixed..

^Ph^LMoO_2_ (**1b**): A single mol­ecule of ^Ph^LMoO_2_ is hydrogen bonded to one disordered DMF mol­ecule, as shown in Fig. 5 ▸, with a distance of 2.03 Å for O11⋯H008 (Table 2 ▸). A second hydrogen bond interaction is between O9—H00*D* with a distance of 2.16 (3) Å. Corresponding hydrogen bond distances in the second molecule in the unit cell are similar. There are three formula units within the contents of the unit cell. Perpendic­ular π-stacking between ^Ph^LMoO_2_ mol­ecules is observed between C5 and the aryl ring centroid (C35–C39) with a distance of 4.597 Å.

**Figure 6:**
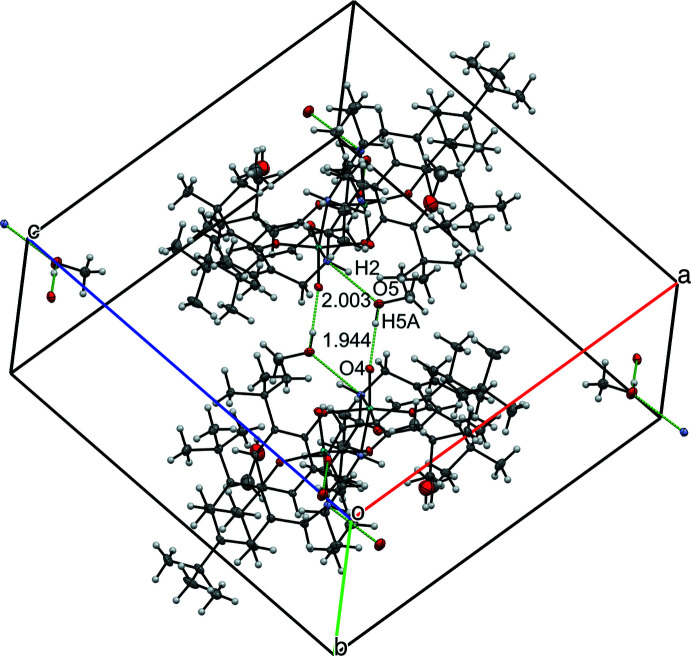
View of four mol­ecules of ^cy^LMoO_2_ and six mol­ecules of methanol in the unit cell with 50% probability ellipsoids, highlighting inter­molecular distances. Distances between H atoms are listed without standard deviations because the H atoms were positionally fixed.

^Cy^LMoO_2_ (**2b**): There are four mol­ecules of ^Cy^LMoO_2_ in the unit cell of this system and the complex is stabilized *via* hydrogen bonding to the solvent MeOH mol­ecule (1.94 Å for O4⋯H5*A* and 2.00 Å for O5⋯H2; Table 3 ▸), as seen in Fig. 6 ▸. There is no indication that there are π-stacking inter­actions between the two mol­ecules. In comparing the hydrogen bonding with the previously reported structure, the main difference is the formation of hydrogen-bonded tetra­mers containing two mol­ecules of **2b** and two mol­ecules of methanol in the current structure. The previously reported structure had one resolved mol­ecule of methanol and one disordered oxygen atom, which form a hydrogen-bonded trimer with one mol­ecule of ^Cy^LMoO_2_ (Ziegler *et al.*, 2009 ▸).

## Database survey

A database search of the Cambridge Structural Database (CSD; Groom *et al.*, 2016 ▸) (webCSD accessed September 22, 2021) and *SciFinder* (SciFinder, 2021 ▸) did not yield any exact matches to the crystal structure for ^Ph^LMoO_2_ (**1b**). There was a similar crystal structure found with the imine form of the ligand (Salen)MoO_2_. A search for ^Cy^LMoO_2_ (**2b**) in the CSD (webCSD accessed September 22, 2021) shows that there is a known structure of the mol­ecule with a different unit cell with accession code HUWGOW (Ziegler *et al.*, 2009 ▸). The *SciFinder* search resulted in the same sources being found. The current structure for ^Cy^LMoO_2_ (**2b**) was solved in space group *P* 2_1_/*n* compared with *P*3_1_ for HUWGOW. The primary additional differences in the structures is an improved *R*1 of 2.78% and more clearly resolved methanol solvent, as compared to the previously reported *R*1 of 5.5% and more disordered methanol solvent (Ziegler *et al.*, 2009 ▸).

## Synthesis and crystallization

**Figure 7:**
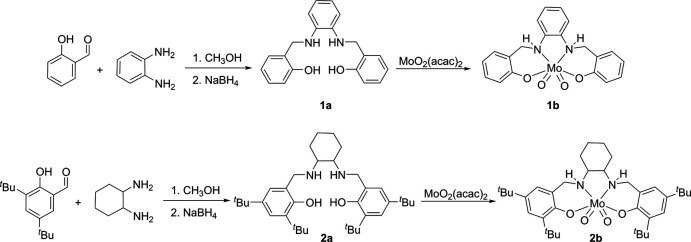
Synthesis of the dioxomolybdenum complexes **1b** and **2b**.

The salan ligands used for stabilizing [MoO_2_]^2+^ in the complexes ^Ph^LMoO_2_ (**1b**) (Rajan *et al.* 1983 ▸) and ^Cy^LMoO_2_ (**2b**) (Ziegler *et al.*, 2009 ▸) were synthesized by the reductive amination of the corresponding salicyl­aldehyde and di­amine. The ligands ^Ph^LH_2_ (**1a**) and ^Cy^LH_2_ (**2a**) were synthesized as off-white solids in 86% and 58% yields, respectively. The reaction scheme is shown in Fig. 7 ▸. Both ligands were successfully characterized by NMR and IR spectroscopy. A salient feature in the ^1^H NMR spectra of both ligands as compared to the precursor salen compounds was the disappearance of the aldimine peak (∼8.50 ppm) and the appearance of the benzylic resonances ∼4.00 ppm. The molybdenum complexes ^Ph^LMoO_2_ (**1b**) and ^Cy^LMoO_2_ (**2b**) were synthesized in 86% and 42% yields, respectively, by the reaction of the corresponding ligands with MoO_2_(acac)_2_ in methanol or aceto­nitrile as solvent. Complexes **1b** and **2b** were also characterized by NMR and IR spectroscopy. Both complexes exhibited stretches {[(Mo=O) = 916 and 876 cm ^−1^(**1b**); 903 and 875 cm^−1^ (**2b**)] characteristic of a *cis*-dioxo molybdenum core in the IR spectrum.

**Procedure for synthesis of ligands**

^Ph^LH_2_ (**1a**): To a solution of 1,2-phenyl­enedi­amine (0.764 g, 7.20 mmol) in methanol (*ca* 7 ml) was added a solution of salicyl­aldehyde (1.76 ml, 14.9 mmol) in methanol (*ca* 8 ml). The mixture was stirred for 6 h at room temperature. The orange precipitate that formed during this period was filtered and washed with methanol, then dried under high vacuum to yield the salophen product as an orange solid (2.19 g, 98%).^1^H NMR (CDCl_3_, 400 MHz, 300 K) *δ* 13.0 (*s*, 2H), 8.63 (*s*, 2H), 7.38 (*d*, ^3^ *J*_H*H*_ = 8 Hz, 2H), 7.35–7.33 (*m*, 2H), 7.26–7.22 (*m*, 2H), 7.05 (*d*, ^3^ *J*_H*H*_ = 8 Hz, 2H), 6.92 (*t*, ^3^ *J*_H*H*_ = 8 Hz, 2H).

To a mixture of methanol (*ca*. 8 ml) and diethyl ether (*ca* 8 ml), was added salophen (1.52 g, 4.81 mmol) followed by NaBH_4_ (1.67 g, 44.4 mmol), and the reaction mixture was stirred at room temperature for 1 h. When the yellow color of the solution changed to colorless, it was transferred into a separatory funnel and DI H_2_O (*ca* 15 ml) was added followed by ethyl acetate (2 × *ca* 15 ml) for extraction. The organic solution was separated and combined, then washed with saturated NaCl solution (*ca* 20 ml). The organic layer was dried over anhydrous Na_2_SO_4_ and filtered. The filtrate was concentrated under vacuum to give a light-yellow solid, which was dried under high vacuum. The color of the solid changed to light brown after 2 h under high vacuum to yield the product (1.32 g, 86%).^1^H NMR (CDCl_3_, 400 MHz, 301 K) *δ* 7.24–7.19 (*m*, 4H), 6.96–6.94 (*m*, 4H), 6.89 (*t*, ^3^ *J*_H*H*_ = 8 Hz, 2H), 6.86 (*t*, ^3^ *J*_H*H*_ = 8 Hz, 2H), 4.40 (*s*, 4H).

^Cy^LH_2_ (**2a**): A 100mL round-bottom flask was charged with *trans-*1,2-di­amino­cyclo­hexane (0.448 g, 4.38 mmol), methanol (*ca*. 16 mL), and 3,5-di-*tert*-butyl­salicyl­aldehyde (2.05 g, 17.5 mmol). The solution was stirred for 24 h at room temperature. The solution resulted in a bright-yellow precipitate. The precipitate was then collected by gravity filtration and washed with cold methanol. The precipitate was dried under high vacuum to remove any residual solvent and yield the salen product (3.85 g, 81%). ^1^H NMR (CDCl_3_, 400 MHz, 301 K) *δ* 13.6 (*br*, 2H), 8.33 (*s*, 2H), 7.34 (*s*, 2H), 7.02 (*s*, 2H), 3.37 (*br*, 2H), 1.98–1.77 (*m*, 4H), 1.40 (*s*, 18H), 1.33–1.29 (*m*, 4H), 1.24 (*s*, 18H).

A 100mL round-bottom flask was charged with the salen product (1.00 g, 2.00 mmol), methanol (*ca* 3 mL), and THF (*ca* 25 mL). NaBH_4_ (9 equivalents) was slowly added into the reaction mixture until the solution was colorless. The reaction was quenched with DI water (*ca* 20 mL), and the product was extracted with ethyl acetate (2 × *ca* 10 ml) using a separatory funnel. The combined organic layers were dried using anhydrous Na_2_SO_4_ and was concentrated under vacuum using the rotary evaporator. The product was then put under high vacuum overnight to ensure it was completely dry (0.577 g, 58%). ^1^H NMR (CDCl_3_, 400 MHz, 301 K) *δ* 7.22 (*d*, ^4^ *J*_H*H*_ = 4 Hz, 2H), 6.87 (*d*, ^4^ *J*_H*H*_ = 4 Hz, 2H), 4.05 (*d*, ^2^ *J*_H*H*_ = 16 Hz, 2H), 3.90 (*d*, ^2^ *J*_H*H*_ = 16 Hz, 2H), 2.51 (*br*, 2H), 2.19 (*br*, 2H), 1.72 (*br*, 2H), 1.44–1.41 (m, 2H), 1.38 (*s*, 18H), 1.28 (*s*, 18H), 1.23–1.20 (*m*, 4H).

**Procedure for synthesis of molybdenum complexes**

Dioxido[2,2′-{l,2-phenyl­enebis(imino­methyl­ene)}bis­(phen­o­lato)]molybdenum(VI) (^Ph^LMoO_2_, **1b**): To a solution of **1a** (1.04 g, 3.29 mmol) in aceto­nitrile (*ca* 20 ml) was added MoO_2_(acac)_2_ (1.07 g, 3.30 mmol) and the mixture was stirred at room temperature for 10 min. The yellow precipitate that formed was filtered and then dried under vacuum to yield the complex as yellow solid (1.24 g, 86%).^1^H NMR (DMSO-*d*_6_, 400 MHz, 301 K) *δ* 7.55 (*d*, ^3^ *J*_H*H*_ = 8 Hz, 1H), 7.37–7.35 (*m*, 1H), 7.19–7.10 (*m*, 4H), 7.07–7.05 (*m*, 1H), 7.02–6.98 (*m*, 2H), 6.91 (*d*, ^3^ *J*_H*H*_ = 8 Hz, 1H), 6.85–6.83 (*m*, 1H), 6.80 (*d*, ^3^ *J*_H*H*_ = 8 Hz, 1H), 6.76–6.68 (*m*, 2H), 6.63 (*d*, ^3^ *J*_H*H*_ = 8 Hz, 1H), 6.59 (*d*, ^3^ *J*_H*H*_ = 8 Hz, 1H), 6.42 (*d*, ^2^ *J*_H*H*_ = 12 Hz, 1H), 5.24 (*d*, ^2^ *J*_H*H*_ = 16 Hz, 1H), 5.16 (*d*, ^2^ *J*_H*H*_ = 16 Hz, 1H), 4.94 (*d*, ^2^ *J*_H*H*_ = 16 Hz, 1H), 4.20 (*d*, ^2^ *J*_H*H*_ = 12 Hz, 1H). ^13^C{^1^H} NMR (DMSO-*d*_6_, 100 MHz, 301 K) *δ* 163.0, 160.2, 155.6, 148.0, 141.1, 130.5, 129.1, 129.0, 128.9, 128.0, 127.9, 125.9, 124.3, 122.9, 120.1, 119.2, 119.1, 118.9, 117.8, 115.3, 111.1, 53.7, 53.6. Selected IR (cm^−1^): 3127 υ(2° N—H); 916, 876 υ(Mo=O).

Crystals of ^Ph^LMoO_2_, **1b** were grown by forming a supersaturated solution of the complex in DMF and layering with hexa­nes. The solution was placed in a refrigerator at 268 K for 1.5 months. Orange–yellow crystals were observed to grow and were collected for structural determination.

(6,6′-{[(Cyclo­hexane-1,2-di­yl)bis­(aza­nedi­yl)]bis­(methyl­ene)}bis­(2,4-di-*tert*-butyl­phenolato))dioxidomolybdenum(VI) (^Cy^LMoO_2_, **2b**): A round-bottom flask equipped with a magnetic stirring bar was charged with MoO_2_(acac)_2_ (0.165 g, 0.506 mmol) and methanol (*ca*. 10 mL). The solution was stirred, and **2a** (0.27 g, 0.51 mmol) was added to the MoO_2_(acac)_2_ dissolved in methanol. The solution was stirred overnight when it turned orange. The solution was filtered, and the solvent removed by evaporation under vacuum to obtain an orange precipitate. The precipitate was triturated with methanol, producing an orange solid, which was separated by gravity filtration and was washed twice with cold methanol (0.108 g, 42%). ^1^H NMR (CDCl_3_, 400 MHz, 301 K) *δ* 7.26 (*s*, 2H), 6.86 (*s*, 2H), 5.28 (*d*, ^2^ *J*_H*H*_ = 16 Hz, 2H), 4.18 (*d*, ^2^ *J*_H*H*_ = 12 Hz, 2H), 2.34–2.28 (*m*, 4H), 1.43 (*s*, 18H), 1.30 (*s*, 18H), 1.19–1.17 (*m*, 4H), 0.88–0.85 (*m*, 4H). ^13^C{^1^H} NMR (CDCl_3_, 100 MHz, 301 K) *δ* 157.1, 152.1, 142.8, 142.3, 142.0, 138.0, 137.7, 137.6, 125.7, 125.4, 124.1, 124.0, 123.0, 122.9, 120.0, 119.6, 65.19, 58.9, 57.6, 53.4, 50.9, 50.5, 35.2, 35.1, 34.3, 34.2, 33.0, 31.6, 31.6, 31.5, 29.9, 29.9, 28.9, 24.5, 24.3, 24.1. Selected IR (cm^−1^): 903, 875 υ(Mo=O).

Crystals of ^Cy^LMoO_2_, **2b** were grown by using a supersaturated solution of the complex dissolved in methanol and allowed to undergo slow evaporation over 2 d. A similar vial was also refrigerated where crystals were seen to form as well. The crystals from the slow evaporation set up were cropped and the orange–yellow crystals were used for structure determination.

## Refinement

Crystal data, data collection, and refinement details are listed in Table 4 ▸. Hydrogen atoms were placed at ideal positions with C—H distances at 0.95 for CH and 0.99 Å for *sp^3^* CH_2_ and CH_3_ using HFIX commands, and refined using a riding model with *U*_iso_(H) = 1.2*U*_eq_(C) for CH, CH_2_, and CH_3_. The structure for ^Ph^MoO_2_ (**1b**) was initially refined in the trigonal crystal system *P*3_2_21; however, this resulted in the solvent DMF having a high level of disorder with many *checkCIF* errors.

## Supplementary Material

Crystal structure: contains datablock(s) 2b, 1b. DOI: 10.1107/S2056989022000524/tx2046sup1.cif

Structure factors: contains datablock(s) 2b. DOI: 10.1107/S2056989022000524/tx20462bsup3.hkl

Structure factors: contains datablock(s) 1b. DOI: 10.1107/S2056989022000524/tx20461bsup2.hkl

CCDC references: 2142074, 2142073

Additional supporting information:  crystallographic information; 3D view; checkCIF report

## Figures and Tables

**Table 1:** Comparison of bond angles (°) between ^Cy^LMoO_2_ (**2b**) with *R*1 of 2.78% and reported structure from Ziegler *et al.* (2009 ▸) with *R*1 of 5.5%

**2b**	Angle	Reported*^*a*^*	Angle
O4—Mo01—O2	100.19 (5)	O2—Mo1—O62	94.3 (2)
O2—Mo01—N2	78.91 (4)	O62—Mo1—N2	86.4 (2)
N1—Mo01—N2	72.40 (4)	N5—Mo1—N2	72.0 (2)
O1—Mo01—N1	76.73 (4)	N5—Mo1—O12	82.7 (2)
O3—Mo01—O1	96.36 (5)	O12—Mo1—O1	93.8 (2)
O3—Mo01—O4	108.55 (5)	O2—Mo1—O1	107.6 (2)

Note: (*a*) Ziegler *et al.* (2009 ▸).

**Table 2:** Hydrogen-bond geometry (Å, °) for **1b** [Chem scheme1]

*D*—H⋯*A*	*D*—H	H⋯*A*	*D*⋯*A*	*D*—H⋯*A*
N2—H008⋯O11	1.00	2.03	2.958 (2)	154
N4—H009⋯O10	1.00	1.99	2.924 (3)	154
N1—H00*D*⋯O12	0.85 (3)	2.15 (3)	2.949 (3)	157 (2)
N3—H00*E*⋯O9	0.79 (3)	2.16 (3)	2.885 (3)	154 (3)

**Table 3:** Hydrogen-bond geometry (Å, °) for **2b** [Chem scheme1]

*D*—H⋯*A*	*D*—H	H⋯*A*	*D*⋯*A*	*D*—H⋯*A*
N2—H2⋯O5^i^	1.00	2.00	2.9319 (16)	153
O5—H5*A*⋯O4	0.84	1.94	2.7837 (16)	177

Symmetry code: (i) 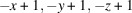 .

**Table 4:** Experimental details

	**1b**	**2b**
Crystal data		
Chemical formula	[Mo(C_20_H_18_N_2_O_2_)O_2_]·2C_3_H_7_NO	[Mo(C_36_H_56_N_2_O_2_)O_2_]·2CH_4_O
*M*_r_	592.49	740.84
Crystal system, space group	Triclinic, *P* 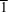	Monoclinic, *P*2_1_/*n*
Temperature (K)	100	105
*a*, *b*, *c* (Å)	9.601, 12.860, 21.428	18.4889 (14), 10.9722 (8), 19.1517 (14)
α, β, γ (°)	91.44, 91.49, 93.22	90, 94.035 (2), 90
*V* (Å^3^)	2639.8	3875.6 (5)
*Z*	4	4
Radiation type	Mo *K*α	Mo *K*α
μ (mm^−1^)	0.54	0.38
Crystal size (mm)	0.34 × 0.29 × 0.29	0.2 × 0.18 × 0.1

Data collection		
Diffractometer	Bruker APEXII CCD	Bruker APEXII CCD
Absorption correction	Multi-scan (*SADABS*; Bruker, 2016 ▸)	Multi-scan (*SADABS*; Bruker, 2016 ▸)
*T*_min_, *T*_max_	0.664, 0.737	0.672, 0.750
No. of measured, independent and observed [*I* > 2σ(*I*)] reflections	146655, 7625, 6364	29075, 9532, 8724
*R*_int_	0.056	0.026
(sin θ/λ)_max_ (Å^−1^)	0.641	0.667

Refinement		
*R*[*F* ^2^ > 2σ(*F* ^2^)], *wR*(*F* ^2^), *S*	0.035, 0.065, 1.06	0.028, 0.070, 1.07
No. of reflections	7625	9532
No. of parameters	683	440
H-atom treatment	H atoms treated by a mixture of independent and constrained refinement	H-atom parameters constrained
Δρ_max_, Δρ_min_ (e Å^−3^)	0.35, −0.38	0.52, −0.52

Computer programs: *APEX2* and *SAINT* (Bruker, 2016 ▸), *SHELXT* (Sheldrick, 2015 ▸), *SHELXL* (Sheldrick, 2008 ▸), and *OLEX2* (Dolomanov *et al.*, 2009 ▸).

## supplementary crystallographic information

### (6,6'-{[(Cyclohexane-1,2-diyl)bis(azanediyl)]bis(methylene)}bis(2,4-di-tert-butylphenolato))dioxidomolybdenum(VI) methanol disolvate (2b) Crystal data

**Table:**

[Mo(C_36_H_56_N_2_O_2_)O_2_]·2CH_4_O	*F*(000) = 1584
*M**_r_* = 740.84	*D*_x_ = 1.270 Mg m^−^^3^
Monoclinic, *P*2_1_/*n*	Mo *K*α radiation, λ = 0.71073 Å
*a* = 18.4889 (14) Å	Cell parameters from 9945 reflections
*b* = 10.9722 (8) Å	θ = 5.3–51.4°
*c* = 19.1517 (14) Å	µ = 0.38 mm^−^^1^
β = 94.035 (2)°	*T* = 105 K
*V* = 3875.6 (5) Å^3^	Prism, clear yellow
*Z* = 4	0.2 × 0.18 × 0.1 mm

### (6,6'-{[(Cyclohexane-1,2-diyl)bis(azanediyl)]bis(methylene)}bis(2,4-di-tert-butylphenolato))dioxidomolybdenum(VI) methanol disolvate (2b) Data collection

**Table:**

Bruker APEXII CCD diffractometer	8724 reflections with *I* > 2σ(*I*)
φ and ω scans	*R*_int_ = 0.026
Absorption correction: multi-scan (SADABS; Bruker, 2016)	θ_max_ = 28.3°, θ_min_ = 5.3°
*T*_min_ = 0.672, *T*_max_ = 0.750	*h* = −24→24
29075 measured reflections	*k* = −14→14
9532 independent reflections	*l* = −25→25

### (6,6'-{[(Cyclohexane-1,2-diyl)bis(azanediyl)]bis(methylene)}bis(2,4-di-tert-butylphenolato))dioxidomolybdenum(VI) methanol disolvate (2b) Refinement

**Table:**

Refinement on *F*^2^	Primary atom site location: dual
Least-squares matrix: full	Hydrogen site location: inferred from neighbouring sites
*R*[*F*^2^ > 2σ(*F*^2^)] = 0.028	H-atom parameters constrained
*wR*(*F*^2^) = 0.070	*w* = 1/[σ^2^(*F*_o_^2^) + (0.0277*P*)^2^ + 2.9594*P*] where *P* = (*F*_o_^2^ + 2*F*_c_^2^)/3
*S* = 1.07	(Δ/σ)_max_ = 0.002
9532 reflections	Δρ_max_ = 0.52 e Å^−^^3^
440 parameters	Δρ_min_ = −0.52 e Å^−^^3^
0 restraints	

### (6,6'-{[(Cyclohexane-1,2-diyl)bis(azanediyl)]bis(methylene)}bis(2,4-di-tert-butylphenolato))dioxidomolybdenum(VI) methanol disolvate (2b) Special details

**Table:**

Geometry. All esds (except the esd in the dihedral angle between two l.s. planes) are estimated using the full covariance matrix. The cell esds are taken into account individually in the estimation of esds in distances, angles and torsion angles; correlations between esds in cell parameters are only used when they are defined by crystal symmetry. An approximate (isotropic) treatment of cell esds is used for estimating esds involving l.s. planes.

### (6,6'-{[(Cyclohexane-1,2-diyl)bis(azanediyl)]bis(methylene)}bis(2,4-di-tert-butylphenolato))dioxidomolybdenum(VI) methanol disolvate (2b) Fractional atomic coordinates and isotropic or equivalent isotropic displacement parameters (Å^2^)

**Table:**

	*x*	*y*	*z*	*U*_iso_*/*U*_eq_	
Mo01	0.59832 (2)	0.45373 (2)	0.68071 (2)	0.00996 (4)	
O1	0.67906 (5)	0.43892 (9)	0.62159 (5)	0.01218 (19)	
O3	0.64642 (6)	0.52033 (10)	0.75027 (6)	0.0154 (2)	
O2	0.51926 (5)	0.39987 (10)	0.73535 (5)	0.01299 (19)	
O4	0.55503 (6)	0.56653 (10)	0.63112 (6)	0.0166 (2)	
N2	0.54408 (6)	0.30514 (11)	0.60679 (6)	0.0124 (2)	
H2	0.562452	0.317877	0.559459	0.015*	
N1	0.64967 (6)	0.26557 (11)	0.71382 (6)	0.0108 (2)	
H1	0.621681	0.233132	0.752592	0.013*	
O5	0.44789 (6)	0.68127 (12)	0.54536 (6)	0.0239 (3)	
H5A	0.481126	0.646691	0.570127	0.036*	
C22	0.46379 (8)	0.32549 (14)	0.59954 (7)	0.0142 (3)	
H22A	0.442308	0.276831	0.559782	0.017*	
H22B	0.453789	0.412566	0.589333	0.017*	
C3	0.84869 (7)	0.31009 (13)	0.70189 (7)	0.0111 (3)	
H3	0.864314	0.255829	0.738559	0.013*	
C5	0.87410 (7)	0.43712 (13)	0.60581 (7)	0.0121 (3)	
H5	0.908405	0.472482	0.577181	0.015*	
C24	0.46022 (7)	0.32631 (13)	0.73127 (7)	0.0119 (3)	
C1	0.75049 (7)	0.41320 (13)	0.63556 (7)	0.0102 (2)	
C25	0.43157 (7)	0.28483 (13)	0.79316 (7)	0.0126 (3)	
C2	0.77461 (7)	0.33656 (13)	0.69077 (7)	0.0108 (2)	
C33	0.46569 (8)	0.32001 (14)	0.86602 (7)	0.0142 (3)	
C4	0.89997 (7)	0.36090 (13)	0.66085 (7)	0.0118 (3)	
C26	0.36957 (8)	0.21086 (14)	0.78600 (8)	0.0146 (3)	
H26	0.348850	0.184092	0.827336	0.017*	
C23	0.42907 (8)	0.28965 (14)	0.66554 (7)	0.0142 (3)	
C9	0.74008 (9)	0.66304 (14)	0.55600 (9)	0.0201 (3)	
H9A	0.697246	0.641207	0.580670	0.030*	
H9B	0.725631	0.716410	0.516425	0.030*	
H9C	0.775058	0.705636	0.588184	0.030*	
C6	0.80076 (7)	0.46405 (12)	0.59066 (7)	0.0111 (2)	
C15	0.72567 (7)	0.28252 (13)	0.74267 (7)	0.0110 (2)	
H15A	0.745494	0.202582	0.758523	0.013*	
H15B	0.725879	0.336533	0.784094	0.013*	
C7	0.77512 (8)	0.54657 (13)	0.52888 (7)	0.0128 (3)	
C10	0.83846 (8)	0.58516 (15)	0.48592 (8)	0.0170 (3)	
H10A	0.820131	0.636333	0.446633	0.025*	
H10B	0.861715	0.512455	0.467948	0.025*	
H10C	0.873925	0.631277	0.515788	0.025*	
C16	0.64240 (8)	0.17496 (13)	0.65555 (7)	0.0141 (3)	
H16	0.672705	0.202504	0.617289	0.017*	
C21	0.66673 (8)	0.04744 (13)	0.67866 (8)	0.0160 (3)	
H21A	0.642144	0.024197	0.720976	0.019*	
H21B	0.719628	0.047899	0.691019	0.019*	
C27	0.33673 (8)	0.17452 (14)	0.72175 (8)	0.0157 (3)	
C28	0.36837 (8)	0.21471 (14)	0.66180 (8)	0.0162 (3)	
H28	0.347797	0.190093	0.617174	0.019*	
C11	0.98156 (7)	0.33692 (13)	0.67445 (7)	0.0129 (3)	
C36	0.54757 (8)	0.29016 (15)	0.87359 (8)	0.0194 (3)	
H36A	0.555113	0.205208	0.860075	0.029*	
H36B	0.565991	0.302178	0.922347	0.029*	
H36C	0.573525	0.344170	0.843165	0.029*	
C19	0.56900 (10)	−0.04481 (15)	0.59694 (9)	0.0219 (3)	
H19A	0.539944	−0.068313	0.636294	0.026*	
H19B	0.559450	−0.104765	0.558777	0.026*	
C20	0.64927 (9)	−0.04664 (15)	0.62109 (9)	0.0224 (3)	
H20A	0.678364	−0.029062	0.580807	0.027*	
H20B	0.662703	−0.128871	0.638897	0.027*	
C14	1.02015 (9)	0.45629 (15)	0.69599 (9)	0.0222 (3)	
H14A	1.012181	0.516351	0.658365	0.033*	
H14B	1.072247	0.441111	0.704617	0.033*	
H14C	1.000677	0.487651	0.738759	0.033*	
C34	0.43001 (10)	0.25202 (16)	0.92482 (8)	0.0229 (3)	
H34A	0.378309	0.272408	0.923167	0.034*	
H34B	0.453306	0.276340	0.970230	0.034*	
H34C	0.435662	0.163973	0.918539	0.034*	
C12	0.99721 (8)	0.24308 (14)	0.73274 (8)	0.0161 (3)	
H12A	0.979753	0.274088	0.776503	0.024*	
H12B	1.049566	0.228556	0.739032	0.024*	
H12C	0.972342	0.166547	0.720004	0.024*	
C8	0.72060 (9)	0.47859 (16)	0.47828 (8)	0.0193 (3)	
H8A	0.677274	0.458088	0.502541	0.029*	
H8B	0.742917	0.403623	0.462137	0.029*	
H8C	0.706920	0.530810	0.437985	0.029*	
C35	0.45589 (9)	0.45675 (14)	0.87842 (8)	0.0205 (3)	
H35A	0.476047	0.502693	0.840440	0.031*	
H35B	0.481207	0.479742	0.923160	0.031*	
H35C	0.404140	0.475293	0.879611	0.031*	
C17	0.56291 (8)	0.17702 (14)	0.62828 (8)	0.0144 (3)	
H17	0.533304	0.155719	0.668282	0.017*	
C13	1.01268 (8)	0.28886 (15)	0.60759 (8)	0.0186 (3)	
H13A	0.987537	0.213576	0.592770	0.028*	
H13B	1.064551	0.272195	0.616823	0.028*	
H13C	1.005854	0.350083	0.570454	0.028*	
C18	0.54628 (9)	0.08268 (15)	0.57084 (8)	0.0193 (3)	
H18A	0.572711	0.103741	0.529292	0.023*	
H18B	0.493712	0.083263	0.556814	0.023*	
C29	0.26833 (8)	0.09489 (15)	0.71510 (9)	0.0185 (3)	
C30	0.28065 (10)	−0.01727 (16)	0.66956 (10)	0.0261 (4)	
H30A	0.290396	0.009075	0.622240	0.039*	
H30B	0.237252	−0.068785	0.667349	0.039*	
H30C	0.322176	−0.063725	0.690021	0.039*	
C37	0.40015 (9)	0.74080 (18)	0.58931 (9)	0.0263 (4)	
H37A	0.356503	0.767075	0.561340	0.039*	
H37B	0.424456	0.812034	0.611086	0.039*	
H37C	0.386558	0.684354	0.625822	0.039*	
C32	0.24605 (10)	0.04964 (19)	0.78619 (10)	0.0299 (4)	
H32A	0.285986	0.003025	0.809534	0.045*	
H32B	0.203172	−0.002637	0.779172	0.045*	
H32C	0.234725	0.119593	0.815306	0.045*	
C31	0.20611 (9)	0.17193 (17)	0.68148 (12)	0.0314 (4)	
H31A	0.200184	0.245472	0.709543	0.047*	
H31B	0.161139	0.124390	0.679387	0.047*	
H31C	0.217224	0.195183	0.633999	0.047*	
O0AA	0.78475 (9)	0.15803 (16)	0.53746 (9)	0.0475 (4)	
H0AA	0.753457	0.134959	0.506334	0.071*	
C38	0.83042 (13)	0.0590 (2)	0.55795 (13)	0.0447 (5)	
H38A	0.809561	−0.016705	0.538274	0.067*	
H38B	0.834867	0.053270	0.609140	0.067*	
H38C	0.878466	0.071852	0.540644	0.067*	

### (6,6'-{[(Cyclohexane-1,2-diyl)bis(azanediyl)]bis(methylene)}bis(2,4-di-tert-butylphenolato))dioxidomolybdenum(VI) methanol disolvate (2b) Atomic displacement parameters (Å^2^)

**Table:**

	*U*^11^	*U*^22^	*U*^33^	*U*^12^	*U*^13^	*U*^23^
Mo01	0.00740 (6)	0.01008 (6)	0.01251 (6)	0.00059 (4)	0.00143 (4)	0.00055 (4)
O1	0.0078 (4)	0.0146 (5)	0.0143 (5)	0.0017 (4)	0.0018 (4)	0.0034 (4)
O3	0.0134 (5)	0.0137 (5)	0.0190 (5)	−0.0010 (4)	0.0010 (4)	−0.0023 (4)
O2	0.0102 (4)	0.0154 (5)	0.0135 (5)	−0.0020 (4)	0.0023 (4)	−0.0009 (4)
O4	0.0135 (5)	0.0168 (5)	0.0197 (5)	0.0044 (4)	0.0030 (4)	0.0037 (4)
N2	0.0099 (5)	0.0152 (6)	0.0119 (5)	0.0022 (5)	0.0000 (4)	0.0012 (4)
N1	0.0079 (5)	0.0117 (5)	0.0127 (5)	−0.0015 (4)	0.0006 (4)	0.0002 (4)
O5	0.0216 (6)	0.0340 (7)	0.0158 (5)	0.0087 (5)	−0.0007 (4)	0.0025 (5)
C22	0.0102 (6)	0.0187 (7)	0.0133 (6)	−0.0004 (5)	−0.0014 (5)	0.0016 (5)
C3	0.0104 (6)	0.0096 (6)	0.0131 (6)	0.0002 (5)	−0.0002 (5)	0.0003 (5)
C5	0.0101 (6)	0.0122 (6)	0.0144 (6)	−0.0018 (5)	0.0029 (5)	0.0005 (5)
C24	0.0072 (6)	0.0128 (6)	0.0156 (6)	0.0009 (5)	0.0004 (5)	0.0007 (5)
C1	0.0077 (6)	0.0100 (6)	0.0131 (6)	−0.0003 (5)	0.0013 (5)	−0.0009 (5)
C25	0.0104 (6)	0.0125 (6)	0.0148 (6)	0.0023 (5)	0.0006 (5)	0.0009 (5)
C2	0.0094 (6)	0.0099 (6)	0.0131 (6)	−0.0020 (5)	0.0014 (5)	−0.0002 (5)
C33	0.0157 (7)	0.0140 (7)	0.0130 (6)	0.0010 (5)	0.0022 (5)	0.0004 (5)
C4	0.0090 (6)	0.0114 (6)	0.0150 (6)	−0.0002 (5)	0.0010 (5)	−0.0013 (5)
C26	0.0113 (6)	0.0144 (7)	0.0185 (7)	0.0013 (5)	0.0042 (5)	0.0029 (5)
C23	0.0115 (6)	0.0178 (7)	0.0133 (6)	0.0002 (5)	−0.0001 (5)	0.0022 (5)
C9	0.0236 (8)	0.0144 (7)	0.0231 (7)	0.0052 (6)	0.0068 (6)	0.0051 (6)
C6	0.0108 (6)	0.0097 (6)	0.0129 (6)	−0.0004 (5)	0.0018 (5)	0.0004 (5)
C15	0.0083 (6)	0.0126 (6)	0.0120 (6)	−0.0017 (5)	−0.0004 (5)	0.0018 (5)
C7	0.0116 (6)	0.0133 (6)	0.0135 (6)	0.0002 (5)	0.0019 (5)	0.0028 (5)
C10	0.0149 (7)	0.0197 (7)	0.0166 (7)	−0.0022 (6)	0.0035 (5)	0.0055 (6)
C16	0.0140 (6)	0.0130 (7)	0.0150 (6)	0.0000 (5)	−0.0013 (5)	−0.0017 (5)
C21	0.0157 (7)	0.0118 (7)	0.0197 (7)	0.0019 (5)	−0.0041 (5)	−0.0005 (5)
C27	0.0093 (6)	0.0157 (7)	0.0219 (7)	−0.0005 (5)	0.0003 (5)	0.0025 (6)
C28	0.0123 (6)	0.0189 (7)	0.0169 (7)	−0.0008 (6)	−0.0028 (5)	0.0014 (6)
C11	0.0077 (6)	0.0140 (7)	0.0169 (6)	0.0000 (5)	0.0006 (5)	0.0010 (5)
C36	0.0177 (7)	0.0221 (8)	0.0177 (7)	0.0026 (6)	−0.0039 (6)	0.0001 (6)
C19	0.0274 (8)	0.0155 (7)	0.0218 (7)	−0.0022 (6)	−0.0057 (6)	−0.0017 (6)
C20	0.0258 (8)	0.0136 (7)	0.0269 (8)	0.0028 (6)	−0.0046 (6)	−0.0039 (6)
C14	0.0144 (7)	0.0178 (7)	0.0336 (9)	−0.0045 (6)	−0.0042 (6)	−0.0005 (6)
C34	0.0301 (9)	0.0251 (8)	0.0138 (7)	−0.0048 (7)	0.0042 (6)	0.0027 (6)
C12	0.0118 (6)	0.0191 (7)	0.0175 (7)	0.0027 (6)	0.0011 (5)	0.0035 (6)
C8	0.0168 (7)	0.0261 (8)	0.0145 (7)	−0.0049 (6)	−0.0012 (5)	0.0026 (6)
C35	0.0274 (8)	0.0161 (7)	0.0183 (7)	0.0028 (6)	0.0031 (6)	−0.0010 (6)
C17	0.0141 (6)	0.0133 (7)	0.0156 (7)	−0.0007 (5)	−0.0006 (5)	0.0005 (5)
C13	0.0134 (7)	0.0242 (8)	0.0185 (7)	0.0046 (6)	0.0033 (5)	0.0037 (6)
C18	0.0236 (8)	0.0161 (7)	0.0171 (7)	0.0005 (6)	−0.0068 (6)	−0.0025 (6)
C29	0.0103 (6)	0.0180 (7)	0.0270 (8)	−0.0030 (6)	0.0014 (6)	0.0012 (6)
C30	0.0221 (8)	0.0218 (8)	0.0345 (9)	−0.0053 (7)	0.0031 (7)	−0.0032 (7)
C37	0.0202 (8)	0.0358 (10)	0.0230 (8)	−0.0026 (7)	0.0027 (6)	−0.0067 (7)
C32	0.0226 (8)	0.0354 (10)	0.0324 (9)	−0.0141 (8)	0.0084 (7)	0.0008 (8)
C31	0.0135 (7)	0.0230 (9)	0.0563 (12)	−0.0015 (7)	−0.0078 (8)	0.0031 (8)
O0AA	0.0424 (9)	0.0457 (9)	0.0550 (10)	0.0012 (8)	0.0073 (7)	−0.0082 (8)
C38	0.0409 (12)	0.0465 (13)	0.0479 (13)	0.0002 (10)	0.0118 (10)	0.0003 (10)

### (6,6'-{[(Cyclohexane-1,2-diyl)bis(azanediyl)]bis(methylene)}bis(2,4-di-tert-butylphenolato))dioxidomolybdenum(VI) methanol disolvate (2b) Geometric parameters (Å, º)

**Table:**

Mo01—O1	1.9428 (10)	C27—C28	1.396 (2)
Mo01—O3	1.7125 (10)	C27—C29	1.535 (2)
Mo01—O2	1.9484 (10)	C28—H28	0.9500
Mo01—O4	1.7226 (11)	C11—C14	1.534 (2)
Mo01—N2	2.3384 (12)	C11—C12	1.531 (2)
Mo01—N1	2.3412 (12)	C11—C13	1.534 (2)
O1—C1	1.3586 (16)	C36—H36A	0.9800
O2—C24	1.3554 (17)	C36—H36B	0.9800
N2—H2	1.0000	C36—H36C	0.9800
N2—C22	1.4979 (18)	C19—H19A	0.9900
N2—C17	1.4989 (19)	C19—H19B	0.9900
N1—H1	1.0000	C19—C20	1.523 (2)
N1—C15	1.4850 (17)	C19—C18	1.534 (2)
N1—C16	1.4935 (18)	C20—H20A	0.9900
O5—H5A	0.8400	C20—H20B	0.9900
O5—C37	1.421 (2)	C14—H14A	0.9800
C22—H22A	0.9900	C14—H14B	0.9800
C22—H22B	0.9900	C14—H14C	0.9800
C22—C23	1.510 (2)	C34—H34A	0.9800
C3—H3	0.9500	C34—H34B	0.9800
C3—C2	1.4016 (18)	C34—H34C	0.9800
C3—C4	1.3902 (19)	C12—H12A	0.9800
C5—H5	0.9500	C12—H12B	0.9800
C5—C4	1.4029 (19)	C12—H12C	0.9800
C5—C6	1.3982 (19)	C8—H8A	0.9800
C24—C25	1.407 (2)	C8—H8B	0.9800
C24—C23	1.4057 (19)	C8—H8C	0.9800
C1—C2	1.3992 (19)	C35—H35A	0.9800
C1—C6	1.4238 (19)	C35—H35B	0.9800
C25—C33	1.5396 (19)	C35—H35C	0.9800
C25—C26	1.403 (2)	C17—H17	1.0000
C2—C15	1.5117 (18)	C17—C18	1.526 (2)
C33—C36	1.546 (2)	C13—H13A	0.9800
C33—C34	1.538 (2)	C13—H13B	0.9800
C33—C35	1.532 (2)	C13—H13C	0.9800
C4—C11	1.5353 (19)	C18—H18A	0.9900
C26—H26	0.9500	C18—H18B	0.9900
C26—C27	1.391 (2)	C29—C30	1.535 (2)
C23—C28	1.389 (2)	C29—C32	1.533 (2)
C9—H9A	0.9800	C29—C31	1.532 (2)
C9—H9B	0.9800	C30—H30A	0.9800
C9—H9C	0.9800	C30—H30B	0.9800
C9—C7	1.539 (2)	C30—H30C	0.9800
C6—C7	1.5380 (19)	C37—H37A	0.9800
C15—H15A	0.9900	C37—H37B	0.9800
C15—H15B	0.9900	C37—H37C	0.9800
C7—C10	1.537 (2)	C32—H32A	0.9800
C7—C8	1.541 (2)	C32—H32B	0.9800
C10—H10A	0.9800	C32—H32C	0.9800
C10—H10B	0.9800	C31—H31A	0.9800
C10—H10C	0.9800	C31—H31B	0.9800
C16—H16	1.0000	C31—H31C	0.9800
C16—C21	1.526 (2)	O0AA—H0AA	0.8400
C16—C17	1.5250 (19)	O0AA—C38	1.415 (3)
C21—H21A	0.9900	C38—H38A	0.9800
C21—H21B	0.9900	C38—H38B	0.9800
C21—C20	1.528 (2)	C38—H38C	0.9800
			
O1—Mo01—O2	157.49 (4)	C23—C28—H28	119.1
O1—Mo01—N2	84.45 (4)	C27—C28—H28	119.1
O1—Mo01—N1	76.73 (4)	C14—C11—C4	109.40 (12)
O3—Mo01—O1	96.36 (5)	C12—C11—C4	111.84 (12)
O3—Mo01—O2	94.58 (5)	C12—C11—C14	108.51 (12)
O3—Mo01—O4	108.55 (5)	C12—C11—C13	108.34 (12)
O3—Mo01—N2	161.00 (5)	C13—C11—C4	109.83 (11)
O3—Mo01—N1	89.26 (5)	C13—C11—C14	108.87 (13)
O2—Mo01—N2	78.91 (4)	C33—C36—H36A	109.5
O2—Mo01—N1	83.82 (4)	C33—C36—H36B	109.5
O4—Mo01—O1	94.88 (5)	C33—C36—H36C	109.5
O4—Mo01—O2	100.19 (5)	H36A—C36—H36B	109.5
O4—Mo01—N2	90.24 (5)	H36A—C36—H36C	109.5
O4—Mo01—N1	161.21 (5)	H36B—C36—H36C	109.5
N2—Mo01—N1	72.40 (4)	H19A—C19—H19B	108.1
C1—O1—Mo01	132.75 (9)	C20—C19—H19A	109.6
C24—O2—Mo01	141.38 (9)	C20—C19—H19B	109.6
Mo01—N2—H2	107.0	C20—C19—C18	110.46 (13)
C22—N2—Mo01	109.40 (9)	C18—C19—H19A	109.6
C22—N2—H2	107.0	C18—C19—H19B	109.6
C22—N2—C17	112.01 (11)	C21—C20—H20A	109.4
C17—N2—Mo01	113.97 (8)	C21—C20—H20B	109.4
C17—N2—H2	107.0	C19—C20—C21	111.22 (14)
Mo01—N1—H1	107.1	C19—C20—H20A	109.4
C15—N1—Mo01	110.21 (8)	C19—C20—H20B	109.4
C15—N1—H1	107.1	H20A—C20—H20B	108.0
C15—N1—C16	113.23 (11)	C11—C14—H14A	109.5
C16—N1—Mo01	111.79 (8)	C11—C14—H14B	109.5
C16—N1—H1	107.1	C11—C14—H14C	109.5
C37—O5—H5A	109.5	H14A—C14—H14B	109.5
N2—C22—H22A	109.4	H14A—C14—H14C	109.5
N2—C22—H22B	109.4	H14B—C14—H14C	109.5
N2—C22—C23	111.14 (11)	C33—C34—H34A	109.5
H22A—C22—H22B	108.0	C33—C34—H34B	109.5
C23—C22—H22A	109.4	C33—C34—H34C	109.5
C23—C22—H22B	109.4	H34A—C34—H34B	109.5
C2—C3—H3	119.0	H34A—C34—H34C	109.5
C4—C3—H3	119.0	H34B—C34—H34C	109.5
C4—C3—C2	121.94 (13)	C11—C12—H12A	109.5
C4—C5—H5	118.1	C11—C12—H12B	109.5
C6—C5—H5	118.1	C11—C12—H12C	109.5
C6—C5—C4	123.81 (13)	H12A—C12—H12B	109.5
O2—C24—C25	119.56 (12)	H12A—C12—H12C	109.5
O2—C24—C23	119.97 (13)	H12B—C12—H12C	109.5
C23—C24—C25	120.45 (13)	C7—C8—H8A	109.5
O1—C1—C2	121.83 (12)	C7—C8—H8B	109.5
O1—C1—C6	117.81 (12)	C7—C8—H8C	109.5
C2—C1—C6	120.33 (12)	H8A—C8—H8B	109.5
C24—C25—C33	121.85 (13)	H8A—C8—H8C	109.5
C26—C25—C24	117.26 (13)	H8B—C8—H8C	109.5
C26—C25—C33	120.89 (13)	C33—C35—H35A	109.5
C3—C2—C15	116.27 (12)	C33—C35—H35B	109.5
C1—C2—C3	119.70 (12)	C33—C35—H35C	109.5
C1—C2—C15	123.98 (12)	H35A—C35—H35B	109.5
C25—C33—C36	111.60 (12)	H35A—C35—H35C	109.5
C34—C33—C25	111.87 (12)	H35B—C35—H35C	109.5
C34—C33—C36	107.23 (13)	N2—C17—C16	107.91 (12)
C35—C33—C25	109.93 (12)	N2—C17—H17	107.5
C35—C33—C36	108.59 (13)	N2—C17—C18	114.02 (12)
C35—C33—C34	107.46 (13)	C16—C17—H17	107.5
C3—C4—C5	116.99 (12)	C16—C17—C18	112.17 (13)
C3—C4—C11	122.44 (12)	C18—C17—H17	107.5
C5—C4—C11	120.57 (12)	C11—C13—H13A	109.5
C25—C26—H26	118.2	C11—C13—H13B	109.5
C27—C26—C25	123.69 (13)	C11—C13—H13C	109.5
C27—C26—H26	118.2	H13A—C13—H13B	109.5
C24—C23—C22	120.24 (13)	H13A—C13—H13C	109.5
C28—C23—C22	120.05 (13)	H13B—C13—H13C	109.5
C28—C23—C24	119.62 (13)	C19—C18—H18A	109.6
H9A—C9—H9B	109.5	C19—C18—H18B	109.6
H9A—C9—H9C	109.5	C17—C18—C19	110.30 (12)
H9B—C9—H9C	109.5	C17—C18—H18A	109.6
C7—C9—H9A	109.5	C17—C18—H18B	109.6
C7—C9—H9B	109.5	H18A—C18—H18B	108.1
C7—C9—H9C	109.5	C30—C29—C27	110.44 (13)
C5—C6—C1	117.17 (12)	C32—C29—C27	112.47 (13)
C5—C6—C7	121.74 (12)	C32—C29—C30	107.76 (14)
C1—C6—C7	121.09 (12)	C31—C29—C27	108.10 (13)
N1—C15—C2	113.51 (11)	C31—C29—C30	109.91 (14)
N1—C15—H15A	108.9	C31—C29—C32	108.13 (15)
N1—C15—H15B	108.9	C29—C30—H30A	109.5
C2—C15—H15A	108.9	C29—C30—H30B	109.5
C2—C15—H15B	108.9	C29—C30—H30C	109.5
H15A—C15—H15B	107.7	H30A—C30—H30B	109.5
C9—C7—C8	109.99 (13)	H30A—C30—H30C	109.5
C6—C7—C9	110.08 (12)	H30B—C30—H30C	109.5
C6—C7—C8	110.57 (12)	O5—C37—H37A	109.5
C10—C7—C9	107.85 (12)	O5—C37—H37B	109.5
C10—C7—C6	111.63 (12)	O5—C37—H37C	109.5
C10—C7—C8	106.62 (12)	H37A—C37—H37B	109.5
C7—C10—H10A	109.5	H37A—C37—H37C	109.5
C7—C10—H10B	109.5	H37B—C37—H37C	109.5
C7—C10—H10C	109.5	C29—C32—H32A	109.5
H10A—C10—H10B	109.5	C29—C32—H32B	109.5
H10A—C10—H10C	109.5	C29—C32—H32C	109.5
H10B—C10—H10C	109.5	H32A—C32—H32B	109.5
N1—C16—H16	108.7	H32A—C32—H32C	109.5
N1—C16—C21	112.61 (11)	H32B—C32—H32C	109.5
N1—C16—C17	106.26 (11)	C29—C31—H31A	109.5
C21—C16—H16	108.7	C29—C31—H31B	109.5
C17—C16—H16	108.7	C29—C31—H31C	109.5
C17—C16—C21	111.73 (12)	H31A—C31—H31B	109.5
C16—C21—H21A	109.3	H31A—C31—H31C	109.5
C16—C21—H21B	109.3	H31B—C31—H31C	109.5
C16—C21—C20	111.54 (12)	C38—O0AA—H0AA	109.5
H21A—C21—H21B	108.0	O0AA—C38—H38A	109.5
C20—C21—H21A	109.3	O0AA—C38—H38B	109.5
C20—C21—H21B	109.3	O0AA—C38—H38C	109.5
C26—C27—C28	117.04 (13)	H38A—C38—H38B	109.5
C26—C27—C29	122.83 (14)	H38A—C38—H38C	109.5
C28—C27—C29	120.13 (13)	H38B—C38—H38C	109.5
C23—C28—C27	121.89 (14)		
			
Mo01—O1—C1—C2	31.67 (19)	C1—C6—C7—C10	−177.16 (13)
Mo01—O1—C1—C6	−150.18 (10)	C1—C6—C7—C8	−58.64 (17)
Mo01—O2—C24—C25	158.74 (11)	C25—C24—C23—C22	−174.72 (13)
Mo01—O2—C24—C23	−19.8 (2)	C25—C24—C23—C28	1.8 (2)
Mo01—N2—C22—C23	−73.12 (13)	C25—C26—C27—C28	0.2 (2)
Mo01—N2—C17—C16	−38.13 (13)	C25—C26—C27—C29	−179.20 (14)
Mo01—N2—C17—C18	−163.45 (10)	C2—C3—C4—C5	−2.2 (2)
Mo01—N1—C15—C2	−61.41 (13)	C2—C3—C4—C11	177.34 (13)
Mo01—N1—C16—C21	−172.68 (9)	C2—C1—C6—C5	−2.0 (2)
Mo01—N1—C16—C17	−50.05 (12)	C2—C1—C6—C7	178.87 (13)
O1—C1—C2—C3	178.24 (12)	C33—C25—C26—C27	−179.11 (14)
O1—C1—C2—C15	−4.4 (2)	C4—C3—C2—C1	2.1 (2)
O1—C1—C6—C5	179.79 (12)	C4—C3—C2—C15	−175.52 (13)
O1—C1—C6—C7	0.69 (19)	C4—C5—C6—C1	1.9 (2)
O2—C24—C25—C33	−0.4 (2)	C4—C5—C6—C7	−178.97 (13)
O2—C24—C25—C26	178.78 (13)	C26—C25—C33—C36	127.48 (15)
O2—C24—C23—C22	3.8 (2)	C26—C25—C33—C34	7.33 (19)
O2—C24—C23—C28	−179.71 (13)	C26—C25—C33—C35	−111.97 (16)
N2—C22—C23—C24	45.68 (19)	C26—C27—C28—C23	−1.3 (2)
N2—C22—C23—C28	−130.82 (14)	C26—C27—C29—C30	−126.65 (16)
N2—C17—C18—C19	178.60 (13)	C26—C27—C29—C32	−6.2 (2)
N1—C16—C21—C20	172.29 (13)	C26—C27—C29—C31	113.07 (17)
N1—C16—C17—N2	56.94 (14)	C23—C24—C25—C33	178.13 (13)
N1—C16—C17—C18	−176.64 (12)	C23—C24—C25—C26	−2.7 (2)
C22—N2—C17—C16	−163.01 (11)	C6—C5—C4—C3	0.1 (2)
C22—N2—C17—C18	71.67 (16)	C6—C5—C4—C11	−179.40 (13)
C22—C23—C28—C27	176.79 (14)	C6—C1—C2—C3	0.1 (2)
C3—C2—C15—N1	−154.63 (12)	C6—C1—C2—C15	177.52 (13)
C3—C4—C11—C14	−114.88 (15)	C15—N1—C16—C21	62.12 (15)
C3—C4—C11—C12	5.36 (19)	C15—N1—C16—C17	−175.25 (11)
C3—C4—C11—C13	125.68 (14)	C16—N1—C15—C2	64.63 (15)
C5—C4—C11—C14	64.61 (17)	C16—C21—C20—C19	−55.27 (18)
C5—C4—C11—C12	−175.14 (13)	C16—C17—C18—C19	55.56 (18)
C5—C4—C11—C13	−54.82 (17)	C21—C16—C17—N2	−179.87 (11)
C5—C6—C7—C9	−115.97 (15)	C21—C16—C17—C18	−53.46 (17)
C5—C6—C7—C10	3.78 (19)	C28—C27—C29—C30	53.95 (19)
C5—C6—C7—C8	122.30 (14)	C28—C27—C29—C32	174.37 (15)
C24—C25—C33—C36	−53.41 (18)	C28—C27—C29—C31	−66.33 (19)
C24—C25—C33—C34	−173.56 (13)	C20—C19—C18—C17	−57.38 (18)
C24—C25—C33—C35	67.14 (17)	C17—N2—C22—C23	54.25 (15)
C24—C25—C26—C27	1.7 (2)	C17—C16—C21—C20	52.79 (17)
C24—C23—C28—C27	0.3 (2)	C18—C19—C20—C21	57.58 (18)
C1—C2—C15—N1	27.90 (19)	C29—C27—C28—C23	178.18 (14)
C1—C6—C7—C9	63.09 (17)		

### (6,6'-{[(Cyclohexane-1,2-diyl)bis(azanediyl)]bis(methylene)}bis(2,4-di-tert-butylphenolato))dioxidomolybdenum(VI) methanol disolvate (2b) Hydrogen-bond geometry (Å, º)

**Table:**

*D*—H···*A*	*D*—H	H···*A*	*D*···*A*	*D*—H···*A*
N2—H2···O5^i^	1.00	2.00	2.9319 (16)	153
O5—H5*A*···O4	0.84	1.94	2.7837 (16)	177

Symmetry code: (i) −*x*+1, −*y*+1, −*z*+1.

### Dioxido{2,2'-[l,2-phenylenebis(iminomethylene)]bis(phenolato)}molybdenum(VI) dimethylformamide disolvate (1b) Crystal data

**Table:**

[Mo(C_20_H_18_N_2_O_2_)O_2_]·2C_3_H_7_NO	*Z* = 4
*M**_r_* = 592.49	*F*(000) = 1224
Triclinic, *P*1	*D*_x_ = 1.491 Mg m^−^^3^
*a* = 9.601 Å	Mo *K*α radiation, λ = 0.71073 Å
*b* = 12.860 Å	Cell parameters from 9515 reflections
*c* = 21.428 Å	θ = 2.3–49.3°
α = 91.44°	µ = 0.54 mm^−^^1^
β = 91.49°	*T* = 100 K
γ = 93.22°	Plate, yellow
*V* = 2639.8 Å^3^	0.34 × 0.29 × 0.29 mm

### Dioxido{2,2'-[l,2-phenylenebis(iminomethylene)]bis(phenolato)}molybdenum(VI) dimethylformamide disolvate (1b) Data collection

**Table:**

Bruker APEXII CCD diffractometer	6364 reflections with *I* > 2σ(*I*)
φ and ω scans	*R*_int_ = 0.056
Absorption correction: multi-scan (SADABS; Bruker, 2016)	θ_max_ = 27.1°, θ_min_ = 2.7°
*T*_min_ = 0.664, *T*_max_ = 0.737	*h* = −12→12
146655 measured reflections	*k* = −16→16
7625 independent reflections	*l* = −27→27

### Dioxido{2,2'-[l,2-phenylenebis(iminomethylene)]bis(phenolato)}molybdenum(VI) dimethylformamide disolvate (1b) Refinement

**Table:**

Refinement on *F*^2^	Primary atom site location: dual
Least-squares matrix: full	Hydrogen site location: mixed
*R*[*F*^2^ > 2σ(*F*^2^)] = 0.035	H atoms treated by a mixture of independent and constrained refinement
*wR*(*F*^2^) = 0.065	*w* = 1/[σ^2^(*F*_o_^2^) + (0.0214*P*)^2^ + 0.638*P*] where *P* = (*F*_o_^2^ + 2*F*_c_^2^)/3
*S* = 1.06	(Δ/σ)_max_ = 0.002
7625 reflections	Δρ_max_ = 0.35 e Å^−^^3^
683 parameters	Δρ_min_ = −0.38 e Å^−^^3^
0 restraints	

### Dioxido{2,2'-[l,2-phenylenebis(iminomethylene)]bis(phenolato)}molybdenum(VI) dimethylformamide disolvate (1b) Special details

**Table:**

Geometry. All esds (except the esd in the dihedral angle between two l.s. planes) are estimated using the full covariance matrix. The cell esds are taken into account individually in the estimation of esds in distances, angles and torsion angles; correlations between esds in cell parameters are only used when they are defined by crystal symmetry. An approximate (isotropic) treatment of cell esds is used for estimating esds involving l.s. planes.

### Dioxido{2,2'-[l,2-phenylenebis(iminomethylene)]bis(phenolato)}molybdenum(VI) dimethylformamide disolvate (1b) Fractional atomic coordinates and isotropic or equivalent isotropic displacement parameters (Å^2^)

**Table:**

	*x*	*y*	*z*	*U*_iso_*/*U*_eq_	
Mo01	0.90908 (2)	0.88143 (2)	0.23791 (2)	0.01047 (6)	
Mo02	0.40906 (2)	0.38140 (2)	0.26211 (2)	0.01045 (6)	
O5	0.36926 (14)	0.42698 (12)	0.17689 (7)	0.0135 (3)	
O1	0.86930 (14)	0.92696 (12)	0.32311 (7)	0.0134 (3)	
O3	1.00613 (14)	0.98497 (13)	0.21447 (8)	0.0165 (3)	
O8	0.51765 (15)	0.28410 (13)	0.23806 (8)	0.0152 (3)	
O7	0.50605 (15)	0.48499 (13)	0.28555 (8)	0.0169 (3)	
N2	0.72468 (16)	0.77486 (14)	0.26078 (8)	0.0098 (3)	
H008	0.736567	0.755680	0.305460	0.012*	
N4	0.22457 (16)	0.27483 (14)	0.23925 (9)	0.0103 (4)	
H009	0.231199	0.252423	0.194450	0.012*	
O4	1.01781 (15)	0.78420 (13)	0.26191 (8)	0.0152 (3)	
O2	0.87093 (14)	0.83127 (13)	0.15364 (7)	0.0141 (3)	
O6	0.37087 (14)	0.33128 (13)	0.34638 (7)	0.0141 (3)	
N1	0.71375 (17)	0.97852 (14)	0.21773 (9)	0.0101 (3)	
N3	0.21399 (17)	0.47869 (14)	0.28219 (9)	0.0101 (3)	
O9	0.2105 (3)	0.59956 (16)	0.39734 (9)	0.0395 (5)	
O10	0.29034 (18)	0.15986 (15)	0.12529 (8)	0.0235 (4)	
O11	0.79013 (18)	0.65963 (14)	0.37463 (8)	0.0234 (4)	
O12	0.7110 (3)	1.09959 (16)	0.10270 (9)	0.0388 (5)	
C40	0.2694 (2)	0.27959 (17)	0.37595 (10)	0.0133 (4)	
C33	0.09199 (19)	0.32526 (16)	0.24527 (10)	0.0097 (4)	
N6	0.2294 (2)	0.00745 (18)	0.07856 (10)	0.0229 (4)	
C13	0.59214 (19)	0.82531 (17)	0.25477 (10)	0.0100 (4)	
N7	0.7296 (2)	0.50764 (18)	0.42150 (10)	0.0231 (5)	
C2	0.6928 (2)	1.05716 (17)	0.32295 (11)	0.0132 (4)	
C44	0.2062 (2)	0.0933 (2)	0.11184 (11)	0.0201 (5)	
H00P	0.114108	0.101089	0.125731	0.024*	
C20	0.7691 (2)	0.77942 (17)	0.12402 (10)	0.0138 (4)	
C26	0.1928 (2)	0.55726 (17)	0.17703 (11)	0.0133 (4)	
C35	0.1970 (2)	0.20156 (17)	0.34516 (11)	0.0138 (4)	
C32	−0.0270 (2)	0.27625 (17)	0.22922 (10)	0.0124 (4)	
H00T	−0.028382	0.209159	0.209538	0.015*	
C21	0.2697 (2)	0.48024 (18)	0.14646 (11)	0.0140 (4)	
N5	0.2261 (2)	0.77472 (17)	0.40850 (10)	0.0223 (4)	
C47	0.7060 (2)	0.5934 (2)	0.38822 (11)	0.0196 (5)	
H00W	0.612568	0.601350	0.374316	0.024*	
N8	0.7263 (2)	1.27474 (17)	0.09144 (10)	0.0223 (4)	
C12	0.4730 (2)	0.77623 (17)	0.27093 (10)	0.0125 (4)	
H00Y	0.473625	0.710632	0.290319	0.015*	
C39	0.2388 (2)	0.3050 (2)	0.43809 (11)	0.0201 (5)	
H00Z	0.291477	0.360886	0.458964	0.024*	
C8	0.58707 (19)	0.92488 (16)	0.22796 (10)	0.0091 (4)	
C29	−0.0390 (2)	0.47127 (17)	0.28501 (10)	0.0123 (4)	
H011	−0.037498	0.538591	0.304393	0.015*	
C19	0.7389 (2)	0.8050 (2)	0.06185 (12)	0.0203 (5)	
H012	0.791590	0.859381	0.042563	0.024*	
C15	0.6965 (2)	0.70132 (17)	0.15474 (11)	0.0142 (4)	
C28	0.08690 (19)	0.42482 (17)	0.27184 (10)	0.0091 (4)	
C41	0.1618 (3)	0.6874 (2)	0.39361 (12)	0.0283 (6)	
H015	0.068263	0.689541	0.378304	0.034*	
C9	0.4611 (2)	0.97132 (17)	0.21493 (10)	0.0125 (4)	
H016	0.460785	1.037336	0.196026	0.015*	
C1	0.7697 (2)	0.98025 (18)	0.35362 (11)	0.0138 (4)	
C5	0.6467 (3)	1.0132 (2)	0.44922 (12)	0.0256 (6)	
H018	0.630612	0.998654	0.491758	0.031*	
C34	0.2286 (2)	0.17687 (17)	0.27776 (11)	0.0134 (4)	
H01G	0.158999	0.123370	0.260434	0.016*	
H01H	0.322041	0.148400	0.275457	0.016*	
C23	0.1469 (3)	0.5132 (2)	0.05077 (12)	0.0258 (6)	
H01I	0.130229	0.498464	0.007471	0.031*	
C6	0.7459 (2)	0.9580 (2)	0.41654 (12)	0.0204 (5)	
H01B	0.796107	0.906242	0.436774	0.024*	
C50	0.6613 (3)	1.1871 (2)	0.10638 (12)	0.0282 (6)	
H3AA	0.569131	1.190364	0.121161	0.034*	
C25	0.0929 (2)	0.61129 (19)	0.14338 (11)	0.0189 (5)	
H01J	0.041127	0.662013	0.164100	0.023*	
C52	0.6615 (3)	1.3763 (2)	0.09717 (13)	0.0289 (6)	
H4AA	0.655244	1.407393	0.055983	0.043*	
H	0.718693	1.422943	0.125606	0.043*	
HA	0.567704	1.365643	0.113700	0.043*	
C3	0.5933 (2)	1.11153 (19)	0.35649 (12)	0.0194 (5)	
H01F	0.541726	1.162479	0.336215	0.023*	
C36	0.0928 (2)	0.1508 (2)	0.37725 (12)	0.0227 (5)	
H01K	0.038307	0.096025	0.356264	0.027*	
C43	0.1617 (3)	0.8762 (2)	0.40289 (13)	0.0286 (6)	
H01V	0.066608	0.864836	0.385335	0.043*	
H01X	0.217185	0.920818	0.375361	0.043*	
H01	0.158583	0.910086	0.444276	0.043*	
C14	0.7286 (2)	0.67698 (17)	0.22211 (11)	0.0132 (4)	
H01A	0.658777	0.624167	0.236808	0.016*	
H01C	0.822034	0.648551	0.225945	0.016*	
C7	0.7171 (2)	1.07799 (17)	0.25468 (11)	0.0132 (4)	
H01D	0.643980	1.122212	0.238363	0.016*	
H01E	0.808727	1.116005	0.250437	0.016*	
C27	0.2171 (2)	0.57802 (17)	0.24528 (11)	0.0134 (4)	
H01L	0.144556	0.623135	0.260818	0.016*	
H01M	0.308883	0.616118	0.252287	0.016*	
C22	0.2461 (2)	0.4580 (2)	0.08324 (11)	0.0201 (5)	
H01Q	0.296160	0.406416	0.062409	0.024*	
C38	0.1364 (3)	0.2525 (2)	0.46952 (12)	0.0268 (6)	
H01S	0.118153	0.270242	0.511713	0.032*	
C18	0.6362 (3)	0.7523 (2)	0.03063 (12)	0.0273 (6)	
H01N	0.614082	0.767239	−0.011561	0.033*	
C16	0.5928 (2)	0.6508 (2)	0.12279 (12)	0.0221 (5)	
H01O	0.538427	0.597338	0.142117	0.027*	
C17	0.5624 (3)	0.6753 (2)	0.06102 (13)	0.0294 (6)	
H01P	0.487821	0.637301	0.039190	0.035*	
C37	0.0627 (3)	0.1754 (2)	0.43895 (13)	0.0293 (6)	
H01T	−0.010064	0.137342	0.459158	0.035*	
C4	0.5703 (3)	1.0908 (2)	0.41929 (12)	0.0278 (6)	
H01R	0.504234	1.127999	0.441810	0.033*	
C46	0.3651 (3)	−0.0174 (2)	0.05437 (13)	0.0305 (6)	
H1AA	0.357558	−0.026473	0.008834	0.046*	
HB	0.394488	−0.082007	0.072697	0.046*	
HC	0.434179	0.039525	0.065371	0.046*	
C24	0.0704 (3)	0.5906 (2)	0.08077 (12)	0.0271 (6)	
H01U	0.004413	0.627654	0.057686	0.032*	
C42	0.3670 (3)	0.7719 (2)	0.43037 (14)	0.0314 (6)	
H0AA	0.372846	0.789365	0.475180	0.047*	
HD	0.425537	0.822557	0.407903	0.047*	
HE	0.399668	0.701912	0.423092	0.047*	
C51	0.8669 (3)	1.2720 (2)	0.06964 (14)	0.0310 (6)	
H5AA	0.902116	1.203125	0.076874	0.046*	
HF	0.926724	1.325264	0.092367	0.046*	
HG	0.867536	1.285745	0.024869	0.046*	
C11	0.3464 (2)	0.82277 (18)	0.25892 (11)	0.0153 (4)	
H01W	0.261648	0.787886	0.270639	0.018*	
C48	0.6238 (4)	0.4321 (3)	0.43236 (16)	0.0491 (9)	
H2AA	0.613110	0.425094	0.477441	0.074*	
HH	0.647881	0.365190	0.413854	0.074*	
HI	0.535937	0.452815	0.413397	0.074*	
C30	−0.1581 (2)	0.42067 (18)	0.27025 (11)	0.0157 (5)	
H01Y	−0.244489	0.449657	0.279109	0.019*	
C31	−0.1535 (2)	0.32303 (18)	0.24115 (11)	0.0156 (5)	
H01Z	−0.238872	0.286402	0.228821	0.019*	
C49	0.8649 (3)	0.4826 (2)	0.44569 (13)	0.0300 (6)	
H02D	0.933605	0.539377	0.437160	0.045*	
H02E	0.892550	0.417988	0.425468	0.045*	
H02F	0.860619	0.473340	0.490867	0.045*	
C45	0.1239 (4)	−0.0683 (3)	0.06749 (17)	0.0505 (9)	
H02A	0.149070	−0.133015	0.087006	0.076*	
H02B	0.110078	−0.080595	0.022377	0.076*	
H02C	0.037358	−0.045270	0.085225	0.076*	
C10	0.3417 (2)	0.92073 (18)	0.22967 (11)	0.0158 (5)	
H022	0.254780	0.949711	0.220761	0.019*	
H00D	0.711 (3)	0.996 (2)	0.1797 (14)	0.024 (8)*	
H00E	0.207 (3)	0.493 (2)	0.3179 (14)	0.015 (7)*	

### Dioxido{2,2'-[l,2-phenylenebis(iminomethylene)]bis(phenolato)}molybdenum(VI) dimethylformamide disolvate (1b) Atomic displacement parameters (Å^2^)

**Table:**

	*U*^11^	*U*^22^	*U*^33^	*U*^12^	*U*^13^	*U*^23^
Mo01	0.00439 (8)	0.00933 (10)	0.01750 (10)	−0.00061 (6)	0.00009 (6)	−0.00087 (7)
Mo02	0.00428 (8)	0.00942 (10)	0.01748 (10)	−0.00056 (6)	−0.00076 (6)	0.00006 (7)
O5	0.0091 (6)	0.0138 (8)	0.0178 (8)	0.0011 (6)	0.0010 (6)	0.0020 (6)
O1	0.0087 (6)	0.0138 (8)	0.0175 (8)	0.0013 (6)	−0.0016 (6)	−0.0029 (6)
O3	0.0082 (6)	0.0153 (8)	0.0254 (9)	−0.0046 (6)	0.0021 (6)	−0.0019 (7)
O8	0.0081 (6)	0.0142 (8)	0.0235 (8)	0.0008 (6)	0.0000 (6)	0.0017 (7)
O7	0.0084 (6)	0.0169 (8)	0.0248 (9)	−0.0037 (6)	−0.0035 (6)	0.0011 (7)
N2	0.0056 (7)	0.0088 (9)	0.0148 (9)	−0.0011 (7)	−0.0022 (6)	−0.0003 (7)
N4	0.0060 (7)	0.0090 (9)	0.0158 (9)	0.0001 (7)	0.0017 (6)	−0.0022 (7)
O4	0.0076 (6)	0.0140 (8)	0.0237 (8)	0.0006 (6)	−0.0014 (6)	−0.0030 (7)
O2	0.0093 (6)	0.0152 (8)	0.0175 (8)	−0.0041 (6)	0.0021 (6)	−0.0007 (6)
O6	0.0085 (6)	0.0155 (8)	0.0175 (8)	−0.0043 (6)	−0.0038 (6)	0.0003 (6)
N1	0.0086 (7)	0.0100 (9)	0.0115 (9)	−0.0008 (7)	0.0018 (6)	0.0010 (7)
N3	0.0084 (7)	0.0092 (9)	0.0123 (9)	−0.0013 (7)	−0.0025 (7)	−0.0028 (7)
O9	0.0747 (15)	0.0213 (10)	0.0206 (10)	−0.0087 (11)	−0.0054 (10)	−0.0046 (8)
O10	0.0253 (8)	0.0227 (9)	0.0220 (9)	−0.0024 (8)	0.0047 (7)	−0.0052 (7)
O11	0.0260 (8)	0.0214 (9)	0.0221 (9)	−0.0029 (7)	−0.0064 (7)	0.0034 (7)
O12	0.0738 (15)	0.0221 (10)	0.0194 (10)	−0.0091 (10)	0.0019 (10)	0.0024 (8)
C40	0.0110 (8)	0.0126 (10)	0.0165 (11)	0.0010 (8)	−0.0008 (8)	0.0035 (8)
C33	0.0077 (8)	0.0099 (10)	0.0117 (10)	0.0008 (8)	0.0009 (7)	0.0004 (8)
N6	0.0258 (10)	0.0234 (11)	0.0184 (11)	−0.0088 (9)	0.0065 (8)	−0.0060 (9)
C13	0.0089 (8)	0.0097 (10)	0.0114 (10)	0.0014 (8)	−0.0011 (7)	−0.0011 (8)
N7	0.0264 (10)	0.0230 (11)	0.0185 (11)	−0.0091 (9)	−0.0071 (8)	0.0041 (9)
C2	0.0099 (8)	0.0101 (10)	0.0189 (11)	−0.0033 (8)	−0.0023 (8)	−0.0040 (8)
C44	0.0218 (10)	0.0233 (13)	0.0155 (11)	0.0008 (10)	0.0039 (9)	0.0011 (10)
C20	0.0105 (9)	0.0123 (10)	0.0184 (11)	0.0015 (8)	0.0010 (8)	−0.0046 (8)
C26	0.0108 (8)	0.0094 (10)	0.0193 (11)	−0.0032 (8)	0.0011 (8)	0.0030 (8)
C35	0.0109 (8)	0.0099 (10)	0.0206 (11)	0.0000 (8)	−0.0007 (8)	0.0034 (8)
C32	0.0093 (8)	0.0088 (10)	0.0185 (11)	−0.0033 (8)	−0.0011 (8)	−0.0006 (8)
C21	0.0075 (8)	0.0148 (11)	0.0198 (11)	−0.0011 (8)	0.0017 (8)	0.0048 (9)
N5	0.0251 (10)	0.0215 (11)	0.0201 (11)	0.0007 (9)	−0.0033 (8)	−0.0015 (9)
C47	0.0215 (10)	0.0227 (13)	0.0142 (11)	−0.0004 (10)	−0.0039 (9)	−0.0015 (10)
N8	0.0252 (10)	0.0211 (11)	0.0207 (11)	0.0001 (9)	0.0031 (8)	0.0006 (9)
C12	0.0098 (9)	0.0094 (10)	0.0178 (11)	−0.0034 (8)	−0.0002 (8)	−0.0019 (8)
C39	0.0203 (10)	0.0224 (12)	0.0171 (12)	−0.0001 (9)	−0.0017 (9)	0.0001 (10)
C8	0.0065 (8)	0.0087 (10)	0.0118 (10)	−0.0016 (7)	−0.0003 (7)	−0.0011 (8)
C29	0.0104 (8)	0.0101 (10)	0.0163 (11)	0.0009 (8)	0.0005 (8)	−0.0008 (8)
C19	0.0210 (10)	0.0209 (12)	0.0189 (12)	−0.0006 (9)	0.0003 (9)	−0.0007 (10)
C15	0.0107 (8)	0.0102 (10)	0.0216 (11)	0.0003 (8)	0.0002 (8)	−0.0039 (9)
C28	0.0051 (8)	0.0103 (10)	0.0114 (10)	−0.0023 (7)	−0.0014 (7)	−0.0004 (8)
C41	0.0408 (14)	0.0288 (15)	0.0138 (12)	−0.0106 (12)	−0.0012 (11)	−0.0015 (11)
C9	0.0096 (8)	0.0105 (10)	0.0174 (11)	0.0012 (8)	−0.0016 (8)	0.0002 (8)
C1	0.0077 (8)	0.0137 (11)	0.0193 (11)	−0.0017 (8)	−0.0018 (8)	−0.0044 (9)
C5	0.0227 (11)	0.0393 (16)	0.0147 (12)	0.0034 (11)	−0.0009 (9)	−0.0032 (11)
C34	0.0107 (8)	0.0086 (10)	0.0206 (11)	−0.0002 (8)	−0.0011 (8)	0.0003 (9)
C23	0.0234 (11)	0.0392 (16)	0.0148 (12)	0.0030 (11)	−0.0003 (9)	0.0014 (11)
C6	0.0162 (10)	0.0263 (13)	0.0185 (12)	0.0020 (9)	−0.0047 (9)	−0.0004 (10)
C50	0.0402 (14)	0.0292 (15)	0.0138 (12)	−0.0104 (12)	0.0003 (11)	0.0013 (11)
C25	0.0140 (9)	0.0187 (12)	0.0248 (13)	0.0034 (9)	0.0043 (9)	0.0065 (10)
C52	0.0312 (13)	0.0275 (15)	0.0292 (14)	0.0091 (12)	0.0037 (11)	0.0028 (12)
C3	0.0146 (9)	0.0185 (12)	0.0247 (12)	0.0035 (9)	−0.0047 (9)	−0.0071 (10)
C36	0.0204 (11)	0.0192 (12)	0.0281 (13)	−0.0040 (9)	0.0011 (10)	0.0060 (10)
C43	0.0304 (12)	0.0271 (14)	0.0286 (14)	0.0083 (12)	−0.0035 (11)	−0.0032 (11)
C14	0.0109 (8)	0.0079 (10)	0.0208 (11)	0.0000 (8)	0.0007 (8)	−0.0002 (8)
C7	0.0102 (8)	0.0072 (10)	0.0219 (11)	−0.0015 (8)	−0.0005 (8)	−0.0007 (8)
C27	0.0105 (8)	0.0076 (10)	0.0218 (11)	−0.0011 (8)	0.0003 (8)	0.0005 (8)
C22	0.0171 (10)	0.0254 (13)	0.0178 (12)	0.0025 (9)	0.0028 (9)	−0.0017 (10)
C38	0.0296 (12)	0.0329 (15)	0.0179 (12)	0.0003 (11)	0.0042 (10)	0.0028 (11)
C18	0.0309 (12)	0.0313 (15)	0.0188 (12)	−0.0002 (11)	−0.0055 (10)	−0.0051 (11)
C16	0.0185 (10)	0.0188 (12)	0.0281 (13)	−0.0041 (9)	0.0002 (9)	−0.0065 (10)
C17	0.0278 (12)	0.0337 (16)	0.0249 (14)	−0.0030 (12)	−0.0078 (11)	−0.0119 (12)
C37	0.0288 (12)	0.0344 (16)	0.0253 (14)	−0.0025 (12)	0.0078 (11)	0.0125 (12)
C4	0.0217 (11)	0.0379 (16)	0.0242 (13)	0.0118 (11)	−0.0006 (10)	−0.0115 (12)
C46	0.0319 (13)	0.0318 (15)	0.0278 (14)	0.0052 (12)	0.0051 (11)	−0.0110 (12)
C24	0.0222 (11)	0.0377 (16)	0.0230 (13)	0.0120 (11)	0.0008 (10)	0.0114 (12)
C42	0.0288 (13)	0.0324 (16)	0.0326 (15)	0.0062 (12)	−0.0088 (11)	−0.0059 (12)
C51	0.0274 (12)	0.0325 (16)	0.0341 (15)	0.0052 (12)	0.0079 (11)	0.0056 (12)
C11	0.0069 (8)	0.0103 (11)	0.0280 (13)	−0.0050 (8)	0.0018 (8)	−0.0036 (9)
C48	0.0536 (19)	0.051 (2)	0.0385 (19)	−0.0346 (17)	−0.0143 (16)	0.0156 (16)
C30	0.0081 (8)	0.0162 (11)	0.0231 (12)	0.0023 (8)	0.0027 (8)	0.0029 (9)
C31	0.0074 (8)	0.0113 (11)	0.0275 (13)	−0.0050 (8)	−0.0029 (8)	0.0022 (9)
C49	0.0310 (13)	0.0319 (15)	0.0276 (14)	0.0049 (12)	−0.0063 (11)	0.0108 (12)
C45	0.0540 (19)	0.050 (2)	0.043 (2)	−0.0335 (18)	0.0155 (16)	−0.0181 (16)
C10	0.0081 (8)	0.0165 (11)	0.0224 (12)	0.0014 (8)	−0.0033 (8)	−0.0035 (9)

### Dioxido{2,2'-[l,2-phenylenebis(iminomethylene)]bis(phenolato)}molybdenum(VI) dimethylformamide disolvate (1b) Geometric parameters (Å, º)

**Table:**

Mo01—O1	1.9567 (16)	C29—C30	1.311 (3)
Mo01—O3	1.6769 (16)	C19—H012	0.9500
Mo01—N2	2.2493 (17)	C19—C18	1.322 (4)
Mo01—O4	1.7518 (14)	C15—C14	1.512 (3)
Mo01—O2	1.9213 (16)	C15—C16	1.324 (3)
Mo01—N1	2.3475 (16)	C41—H015	0.9500
Mo02—O5	1.9665 (15)	C9—H016	0.9500
Mo02—O8	1.7493 (15)	C9—C10	1.335 (3)
Mo02—O7	1.6423 (17)	C1—C6	1.407 (3)
Mo02—N4	2.2145 (18)	C5—H018	0.9500
Mo02—O6	1.9692 (15)	C5—C6	1.410 (3)
Mo02—N3	2.3529 (16)	C5—C4	1.426 (4)
O5—C21	1.368 (2)	C34—H01G	0.9900
O1—C1	1.377 (2)	C34—H01H	0.9900
N2—H008	1.0000	C23—H01I	0.9500
N2—C13	1.465 (2)	C23—C22	1.401 (3)
N2—C14	1.492 (3)	C23—C24	1.420 (4)
N4—H009	1.0000	C6—H01B	0.9500
N4—C33	1.468 (2)	C50—H3AA	0.9500
N4—C34	1.525 (3)	C25—H01J	0.9500
O2—C20	1.295 (3)	C25—C24	1.370 (4)
O6—C40	1.332 (3)	C52—H4AA	0.9800
N1—C8	1.389 (3)	C52—H	0.9800
N1—C7	1.486 (3)	C52—HA	0.9800
N1—H00D	0.85 (3)	C3—H01F	0.9500
N3—C28	1.379 (3)	C3—C4	1.399 (4)
N3—C27	1.519 (3)	C36—H01K	0.9500
N3—H00E	0.79 (3)	C36—C37	1.393 (4)
O9—C41	1.250 (4)	C43—H01V	0.9800
O10—C44	1.169 (3)	C43—H01X	0.9800
O11—C47	1.187 (3)	C43—H01	0.9800
O12—C50	1.249 (4)	C14—H01A	0.9900
C40—C35	1.338 (3)	C14—H01C	0.9900
C40—C39	1.405 (3)	C7—H01D	0.9900
C33—C32	1.307 (3)	C7—H01E	0.9900
C33—C28	1.392 (3)	C27—H01L	0.9900
N6—C44	1.332 (3)	C27—H01M	0.9900
N6—C46	1.464 (3)	C22—H01Q	0.9500
N6—C45	1.377 (4)	C38—H01S	0.9500
C13—C12	1.333 (3)	C38—C37	1.333 (4)
C13—C8	1.419 (3)	C18—H01N	0.9500
N7—C47	1.355 (3)	C18—C17	1.372 (4)
N7—C48	1.394 (4)	C16—H01O	0.9500
N7—C49	1.443 (3)	C16—C17	1.396 (4)
C2—C1	1.430 (3)	C17—H01P	0.9500
C2—C3	1.415 (3)	C37—H01T	0.9500
C2—C7	1.515 (3)	C4—H01R	0.9500
C44—H00P	0.9500	C46—H1AA	0.9800
C20—C19	1.407 (3)	C46—HB	0.9800
C20—C15	1.380 (3)	C46—HC	0.9800
C26—C21	1.425 (3)	C24—H01U	0.9500
C26—C25	1.410 (3)	C42—H0AA	0.9800
C26—C27	1.490 (3)	C42—HD	0.9800
C35—C34	1.513 (3)	C42—HE	0.9800
C35—C36	1.373 (3)	C51—H5AA	0.9800
C32—H00T	0.9500	C51—HF	0.9800
C32—C31	1.412 (3)	C51—HG	0.9800
C21—C22	1.388 (3)	C11—H01W	0.9500
N5—C41	1.280 (4)	C11—C10	1.424 (3)
N5—C43	1.482 (3)	C48—H2AA	0.9800
N5—C42	1.423 (3)	C48—HH	0.9800
C47—H00W	0.9500	C48—HI	0.9800
N8—C50	1.308 (3)	C30—H01Y	0.9500
N8—C52	1.481 (3)	C30—C31	1.391 (3)
N8—C51	1.442 (3)	C31—H01Z	0.9500
C12—H00Y	0.9500	C49—H02D	0.9800
C12—C11	1.405 (3)	C49—H02E	0.9800
C39—H00Z	0.9500	C49—H02F	0.9800
C39—C38	1.364 (4)	C45—H02A	0.9800
C8—C9	1.403 (2)	C45—H02B	0.9800
C29—H011	0.9500	C45—H02C	0.9800
C29—C28	1.410 (2)	C10—H022	0.9500
			
O1—Mo01—N2	77.65 (7)	O1—C1—C6	118.21 (19)
O1—Mo01—N1	80.23 (6)	C6—C1—C2	120.4 (2)
O3—Mo01—O1	100.51 (8)	C6—C5—H018	119.6
O3—Mo01—N2	161.90 (6)	C6—C5—C4	120.9 (2)
O3—Mo01—O4	109.80 (7)	C4—C5—H018	119.6
O3—Mo01—O2	92.51 (8)	N4—C34—H01G	109.6
O3—Mo01—N1	86.75 (6)	N4—C34—H01H	109.6
N2—Mo01—N1	75.18 (6)	C35—C34—N4	110.35 (17)
O4—Mo01—O1	94.14 (7)	C35—C34—H01G	109.6
O4—Mo01—N2	88.30 (6)	C35—C34—H01H	109.6
O4—Mo01—O2	98.32 (7)	H01G—C34—H01H	108.1
O4—Mo01—N1	163.31 (7)	C22—C23—H01I	119.1
O2—Mo01—O1	157.78 (6)	C22—C23—C24	121.9 (2)
O2—Mo01—N2	84.38 (7)	C24—C23—H01I	119.1
O2—Mo01—N1	82.63 (6)	C1—C6—C5	119.3 (2)
O5—Mo02—N4	81.39 (7)	C1—C6—H01B	120.4
O5—Mo02—O6	158.10 (6)	C5—C6—H01B	120.4
O5—Mo02—N3	81.07 (6)	O12—C50—N8	125.0 (3)
O8—Mo02—O5	94.30 (7)	O12—C50—H3AA	117.5
O8—Mo02—N4	89.52 (7)	N8—C50—H3AA	117.5
O8—Mo02—O6	98.33 (7)	C26—C25—H01J	120.0
O8—Mo02—N3	163.76 (7)	C24—C25—C26	120.0 (2)
O7—Mo02—O5	96.96 (8)	C24—C25—H01J	120.0
O7—Mo02—O8	109.00 (7)	N8—C52—H4AA	109.5
O7—Mo02—N4	161.48 (7)	N8—C52—H	109.5
O7—Mo02—O6	95.73 (8)	N8—C52—HA	109.5
O7—Mo02—N3	87.08 (7)	H4AA—C52—H	109.5
N4—Mo02—N3	74.43 (6)	H4AA—C52—HA	109.5
O6—Mo02—N4	80.91 (7)	H—C52—HA	109.5
O6—Mo02—N3	81.84 (6)	C2—C3—H01F	119.8
C21—O5—Mo02	137.80 (14)	C4—C3—C2	120.5 (2)
C1—O1—Mo01	138.48 (14)	C4—C3—H01F	119.8
Mo01—N2—H008	107.3	C35—C36—H01K	118.3
C13—N2—Mo01	112.59 (12)	C35—C36—C37	123.5 (3)
C13—N2—H008	107.3	C37—C36—H01K	118.3
C13—N2—C14	113.36 (16)	N5—C43—H01V	109.5
C14—N2—Mo01	108.75 (12)	N5—C43—H01X	109.5
C14—N2—H008	107.3	N5—C43—H01	109.5
Mo02—N4—H009	107.0	H01V—C43—H01X	109.5
C33—N4—Mo02	113.03 (13)	H01V—C43—H01	109.5
C33—N4—H009	107.0	H01X—C43—H01	109.5
C33—N4—C34	111.85 (15)	N2—C14—C15	108.70 (16)
C34—N4—Mo02	110.51 (12)	N2—C14—H01A	109.9
C34—N4—H009	107.0	N2—C14—H01C	109.9
C20—O2—Mo01	136.10 (14)	C15—C14—H01A	109.9
C40—O6—Mo02	138.81 (13)	C15—C14—H01C	109.9
Mo01—N1—H00D	110.7 (19)	H01A—C14—H01C	108.3
C8—N1—Mo01	113.88 (13)	N1—C7—C2	110.47 (17)
C8—N1—C7	107.69 (16)	N1—C7—H01D	109.6
C8—N1—H00D	106 (2)	N1—C7—H01E	109.6
C7—N1—Mo01	112.87 (12)	C2—C7—H01D	109.6
C7—N1—H00D	105 (2)	C2—C7—H01E	109.6
Mo02—N3—H00E	113.0 (19)	H01D—C7—H01E	108.1
C28—N3—Mo02	114.65 (13)	N3—C27—H01L	109.1
C28—N3—C27	109.21 (16)	N3—C27—H01M	109.1
C28—N3—H00E	99 (2)	C26—C27—N3	112.45 (18)
C27—N3—Mo02	111.45 (12)	C26—C27—H01L	109.1
C27—N3—H00E	109 (2)	C26—C27—H01M	109.1
O6—C40—C35	118.3 (2)	H01L—C27—H01M	107.8
O6—C40—C39	121.4 (2)	C21—C22—C23	118.6 (2)
C35—C40—C39	120.3 (2)	C21—C22—H01Q	120.7
C32—C33—N4	121.34 (19)	C23—C22—H01Q	120.7
C32—C33—C28	117.14 (18)	C39—C38—H01S	121.2
C28—C33—N4	121.41 (18)	C37—C38—C39	117.7 (2)
C44—N6—C46	124.5 (2)	C37—C38—H01S	121.2
C44—N6—C45	120.4 (2)	C19—C18—H01N	121.0
C45—N6—C46	115.0 (3)	C19—C18—C17	118.0 (2)
C12—C13—N2	120.75 (18)	C17—C18—H01N	121.0
C12—C13—C8	118.66 (17)	C15—C16—H01O	119.6
C8—C13—N2	120.47 (17)	C15—C16—C17	120.7 (3)
C47—N7—C48	121.9 (2)	C17—C16—H01O	119.6
C47—N7—C49	124.1 (2)	C18—C17—C16	122.5 (3)
C48—N7—C49	113.9 (2)	C18—C17—H01P	118.8
C1—C2—C7	119.57 (18)	C16—C17—H01P	118.8
C3—C2—C1	119.5 (2)	C36—C37—H01T	120.1
C3—C2—C7	120.89 (19)	C38—C37—C36	119.7 (2)
O10—C44—N6	125.1 (2)	C38—C37—H01T	120.1
O10—C44—H00P	117.5	C5—C4—H01R	120.3
N6—C44—H00P	117.5	C3—C4—C5	119.5 (2)
O2—C20—C19	118.2 (2)	C3—C4—H01R	120.3
O2—C20—C15	118.5 (2)	N6—C46—H1AA	109.5
C15—C20—C19	123.3 (2)	N6—C46—HB	109.5
C21—C26—C27	119.07 (18)	N6—C46—HC	109.5
C25—C26—C21	120.4 (2)	H1AA—C46—HB	109.5
C25—C26—C27	120.5 (2)	H1AA—C46—HC	109.5
C40—C35—C34	119.71 (19)	HB—C46—HC	109.5
C40—C35—C36	116.4 (2)	C23—C24—H01U	120.4
C36—C35—C34	123.8 (2)	C25—C24—C23	119.3 (2)
C33—C32—H00T	120.0	C25—C24—H01U	120.4
C33—C32—C31	120.0 (2)	N5—C42—H0AA	109.5
C31—C32—H00T	120.0	N5—C42—HD	109.5
O5—C21—C26	122.5 (2)	N5—C42—HE	109.5
O5—C21—C22	117.6 (2)	H0AA—C42—HD	109.5
C22—C21—C26	119.91 (19)	H0AA—C42—HE	109.5
C41—N5—C43	123.2 (2)	HD—C42—HE	109.5
C41—N5—C42	117.1 (2)	N8—C51—H5AA	109.5
C42—N5—C43	119.7 (2)	N8—C51—HF	109.5
O11—C47—N7	126.7 (2)	N8—C51—HG	109.5
O11—C47—H00W	116.6	H5AA—C51—HF	109.5
N7—C47—H00W	116.6	H5AA—C51—HG	109.5
C50—N8—C52	122.4 (2)	HF—C51—HG	109.5
C50—N8—C51	118.5 (2)	C12—C11—H01W	119.1
C51—N8—C52	119.1 (2)	C12—C11—C10	121.85 (19)
C13—C12—H00Y	120.4	C10—C11—H01W	119.1
C13—C12—C11	119.15 (19)	N7—C48—H2AA	109.5
C11—C12—H00Y	120.4	N7—C48—HH	109.5
C40—C39—H00Z	118.8	N7—C48—HI	109.5
C38—C39—C40	122.4 (3)	H2AA—C48—HH	109.5
C38—C39—H00Z	118.8	H2AA—C48—HI	109.5
N1—C8—C13	117.13 (16)	HH—C48—HI	109.5
N1—C8—C9	120.23 (18)	C29—C30—H01Y	121.2
C9—C8—C13	122.55 (18)	C29—C30—C31	117.66 (19)
C28—C29—H011	120.3	C31—C30—H01Y	121.2
C30—C29—H011	120.3	C32—C31—H01Z	118.7
C30—C29—C28	119.4 (2)	C30—C31—C32	122.6 (2)
C20—C19—H012	120.4	C30—C31—H01Z	118.7
C18—C19—C20	119.1 (2)	N7—C49—H02D	109.5
C18—C19—H012	120.4	N7—C49—H02E	109.5
C20—C15—C14	122.4 (2)	N7—C49—H02F	109.5
C16—C15—C20	116.4 (2)	H02D—C49—H02E	109.5
C16—C15—C14	121.2 (2)	H02D—C49—H02F	109.5
N3—C28—C33	115.73 (17)	H02E—C49—H02F	109.5
N3—C28—C29	121.04 (19)	N6—C45—H02A	109.5
C33—C28—C29	123.17 (19)	N6—C45—H02B	109.5
O9—C41—N5	126.1 (3)	N6—C45—H02C	109.5
O9—C41—H015	117.0	H02A—C45—H02B	109.5
N5—C41—H015	117.0	H02A—C45—H02C	109.5
C8—C9—H016	120.7	H02B—C45—H02C	109.5
C10—C9—C8	118.57 (19)	C9—C10—C11	119.12 (18)
C10—C9—H016	120.7	C9—C10—H022	120.4
O1—C1—C2	121.4 (2)	C11—C10—H022	120.4
			
Mo01—O1—C1—C2	−28.3 (3)	C12—C13—C8—C9	−3.3 (3)
Mo01—O1—C1—C6	152.37 (19)	C12—C11—C10—C9	−2.1 (4)
Mo01—N2—C13—C12	−175.03 (17)	C39—C40—C35—C34	178.42 (19)
Mo01—N2—C13—C8	8.9 (2)	C39—C40—C35—C36	0.9 (3)
Mo01—N2—C14—C15	−69.85 (16)	C39—C38—C37—C36	0.5 (4)
Mo01—O2—C20—C19	144.35 (18)	C8—N1—C7—C2	55.7 (2)
Mo01—O2—C20—C15	−36.3 (3)	C8—C13—C12—C11	2.2 (3)
Mo01—N1—C8—C13	6.3 (2)	C8—C9—C10—C11	1.1 (3)
Mo01—N1—C8—C9	−176.91 (16)	C29—C30—C31—C32	2.2 (3)
Mo01—N1—C7—C2	−70.89 (17)	C19—C20—C15—C14	−178.5 (2)
Mo02—O5—C21—C26	28.4 (3)	C19—C20—C15—C16	−1.0 (3)
Mo02—O5—C21—C22	−152.48 (18)	C19—C18—C17—C16	−0.5 (4)
Mo02—N4—C33—C32	174.91 (17)	C15—C20—C19—C18	0.0 (3)
Mo02—N4—C33—C28	−9.1 (2)	C15—C16—C17—C18	−0.6 (4)
Mo02—N4—C34—C35	72.40 (16)	C28—N3—C27—C26	−58.0 (2)
Mo02—O6—C40—C35	37.3 (3)	C28—C33—C32—C31	−2.3 (3)
Mo02—O6—C40—C39	−143.20 (19)	C28—C29—C30—C31	−0.9 (3)
Mo02—N3—C28—C33	−6.2 (2)	C1—C2—C3—C4	0.2 (4)
Mo02—N3—C28—C29	176.63 (15)	C1—C2—C7—N1	48.1 (3)
Mo02—N3—C27—C26	69.68 (18)	C34—N4—C33—C32	−59.6 (3)
O5—C21—C22—C23	−178.6 (2)	C34—N4—C33—C28	116.4 (2)
O1—C1—C6—C5	178.5 (2)	C34—C35—C36—C37	−178.7 (2)
N2—C13—C12—C11	−173.9 (2)	C6—C5—C4—C3	0.5 (4)
N2—C13—C8—N1	−10.4 (3)	C25—C26—C21—O5	178.9 (2)
N2—C13—C8—C9	172.8 (2)	C25—C26—C21—C22	−0.1 (3)
N4—C33—C32—C31	173.9 (2)	C25—C26—C27—N3	130.2 (2)
N4—C33—C28—N3	10.4 (3)	C52—N8—C50—O12	−179.7 (3)
N4—C33—C28—C29	−172.52 (19)	C3—C2—C1—O1	−178.8 (2)
O2—C20—C19—C18	179.4 (2)	C3—C2—C1—C6	0.6 (3)
O2—C20—C15—C14	2.2 (3)	C3—C2—C7—N1	−130.8 (2)
O2—C20—C15—C16	179.62 (19)	C36—C35—C34—N4	124.9 (2)
O6—C40—C35—C34	−2.1 (3)	C43—N5—C41—O9	−180.0 (3)
O6—C40—C35—C36	−179.60 (18)	C14—N2—C13—C12	61.0 (3)
O6—C40—C39—C38	−179.3 (2)	C14—N2—C13—C8	−115.1 (2)
N1—C8—C9—C10	−175.1 (2)	C14—C15—C16—C17	178.7 (2)
C40—C35—C34—N4	−52.4 (2)	C7—N1—C8—C13	−119.7 (2)
C40—C35—C36—C37	−1.3 (3)	C7—N1—C8—C9	57.1 (2)
C40—C39—C38—C37	−0.9 (4)	C7—C2—C1—O1	2.3 (3)
C33—N4—C34—C35	−54.5 (2)	C7—C2—C1—C6	−178.3 (2)
C33—C32—C31—C30	−0.5 (3)	C7—C2—C3—C4	179.1 (2)
C13—N2—C14—C15	56.2 (2)	C27—N3—C28—C33	119.63 (19)
C13—C12—C11—C10	0.3 (3)	C27—N3—C28—C29	−57.5 (3)
C13—C8—C9—C10	1.6 (3)	C27—C26—C21—O5	−2.5 (3)
C2—C1—C6—C5	−0.8 (4)	C27—C26—C21—C22	178.4 (2)
C2—C3—C4—C5	−0.8 (4)	C27—C26—C25—C24	−179.3 (2)
C20—C19—C18—C17	0.8 (4)	C22—C23—C24—C25	−0.8 (4)
C20—C15—C14—N2	53.3 (2)	C16—C15—C14—N2	−124.0 (2)
C20—C15—C16—C17	1.3 (3)	C4—C5—C6—C1	0.3 (4)
C26—C21—C22—C23	0.5 (4)	C46—N6—C44—O10	0.9 (4)
C26—C25—C24—C23	1.1 (4)	C24—C23—C22—C21	−0.1 (4)
C35—C40—C39—C38	0.2 (3)	C42—N5—C41—O9	1.1 (4)
C35—C36—C37—C38	0.6 (4)	C51—N8—C50—O12	−0.8 (4)
C32—C33—C28—N3	−173.44 (19)	C48—N7—C47—O11	−177.0 (3)
C32—C33—C28—C29	3.6 (3)	C30—C29—C28—N3	174.9 (2)
C21—C26—C25—C24	−0.7 (4)	C30—C29—C28—C33	−2.0 (3)
C21—C26—C27—N3	−48.4 (3)	C49—N7—C47—O11	−0.7 (4)
C12—C13—C8—N1	173.4 (2)	C45—N6—C44—O10	177.3 (3)

### Dioxido{2,2'-[l,2-phenylenebis(iminomethylene)]bis(phenolato)}molybdenum(VI) dimethylformamide disolvate (1b) Hydrogen-bond geometry (Å, º)

**Table:**

*D*—H···*A*	*D*—H	H···*A*	*D*···*A*	*D*—H···*A*
N2—H008···O11	1.00	2.03	2.958 (2)	154
N4—H009···O10	1.00	1.99	2.924 (3)	154
N1—H00*D*···O12	0.85 (3)	2.15 (3)	2.949 (3)	157 (2)
N3—H00*E*···O9	0.79 (3)	2.16 (3)	2.885 (3)	154 (3)
